# Unlocking the Diversity of Pyrroloiminoquinones Produced by Latrunculid Sponge Species

**DOI:** 10.3390/md19020068

**Published:** 2021-01-28

**Authors:** Jarmo-Charles J. Kalinski, Rui W. M. Krause, Shirley Parker-Nance, Samantha C. Waterworth, Rosemary A. Dorrington

**Affiliations:** 1 Department of Biochemistry and Microbiology, Rhodes University, Makhanda 6140, South Africa; j.kalinski@ru.ac.za (J.-C.J.K.); shirley@saeon.ac.za (S.P.-N.); swaterworth@wisc.edu (S.C.W.); 2 Department of Chemistry, Rhodes University, Makhanda 6140, South Africa; r.krause@ru.ac.za; 3 South African Environmental Observation Network, Elwandle Coastal Node, Port Elizabeth 6001, South Africa; 4 South African Institute for Aquatic Biodiversity, Makhanda 6140, South Africa; 5 Division of Pharmaceutical Sciences, University of Wisconsin, Madison, WI 53705, USA

**Keywords:** molecular networking, LC-MS-MS, makaluvamine, discorhabdin, tsitsikammamine, chemotype, collision-induced dissociation, odd-electron

## Abstract

Sponges of the Latrunculiidae family produce bioactive pyrroloiminoquinone alkaloids including makaluvamines, discorhabdins, and tsitsikammamines. The aim of this study was to use LC-ESI-MS/MS-driven molecular networking to characterize the pyrroloiminoquinone secondary metabolites produced by six latrunculid species. These are *Tsitsikamma favus*, *Tsitsikamma pedunculata*, *Cyclacanthia bellae,* and *Latrunculia apicalis* as well as the recently discovered species, *Tsitsikamma nguni* and *Tsitsikamma michaeli*. Organic extracts of 43 sponges were analyzed, revealing distinct species-specific chemical profiles. More than 200 known and unknown putative pyrroloiminoquinones and related compounds were detected, including unprecedented makaluvamine-discorhabdin adducts and hydroxylated discorhabdin I derivatives. The chemical profiles of the new species *T. nguni* closely resembled those of the known *T. favus* (chemotype I), but with a higher abundance of tsitsikammamines vs. discorhabdins. *T. michaeli* sponges displayed two distinct chemical profiles, either producing mostly the same discorhabdins as *T. favus* (chemotype I) or non- or monobrominated, hydroxylated discorhabdins. *C. bellae* and *L. apicalis* produced similar pyrroloiminoquinone chemistry to one another, characterized by sulfur-containing discorhabdins and related adducts and oligomers. This study highlights the variability of pyrroloiminoquinone production by latrunculid species, identifies novel isolation targets, and offers fundamental insights into the collision-induced dissociation of pyrroloiminoquinones.

## 1. Introduction

**Figure 1:**
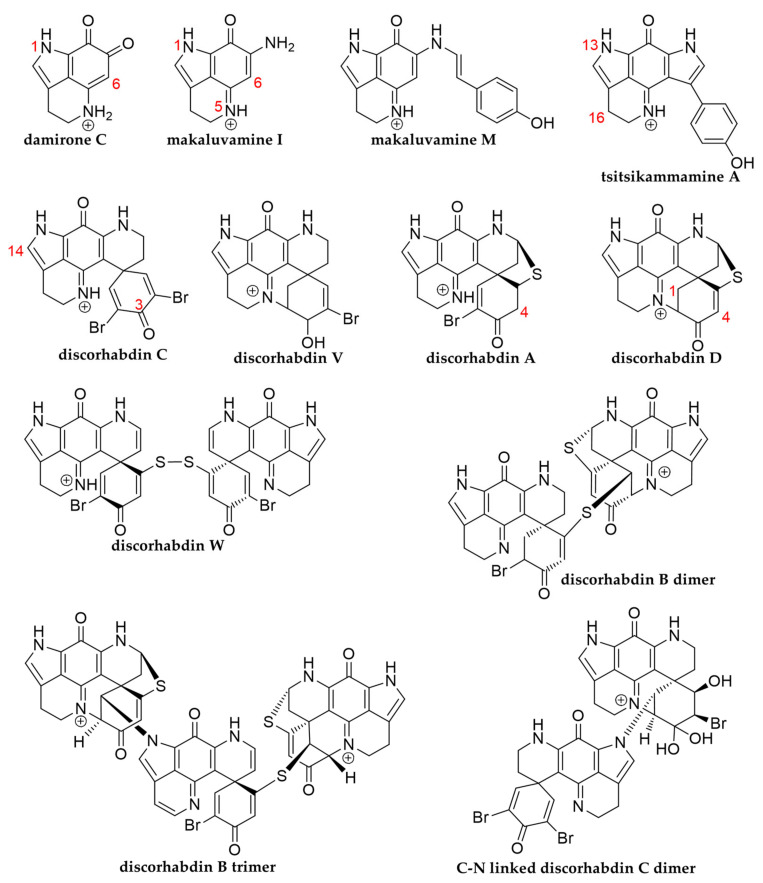
Representative structures encompassing the major pyrroloiminoquinone classes.

The emergence of new and multidrug-resistant pathogens [1,2,3,4] creates an ongoing need to investigate novel compounds from marine organisms, since marine natural products have proven useful for medical and pharmacological applications [5,6] and include potential antimicrobial [7], antiviral [8] and antifungal [9] drug leads. Marine sponges of the family Latrunculiidae are known to be prolific producers of cytotoxic pyrroloiminoquinones, a class of alkaloids with potential as leads in anticancer, antiparasite and antibacterial drug development [10,11,12,13]. In nature, these compounds are mostly encountered as makaluvamines, tsitsikammamines, and discorhabdins, all sharing the characteristic tricyclic pyrroloiminoquinone structural motif and they are often isolated alongside related pyrroloquinolines such as damirones (Figure 1) [12,13]. Makaluvamine structures are comprised of the pyrroloiminoquinone core (≡makaluvamine I without peripheral -NH_2_) with various substituents including *N*-methyl, bromine and *N*-phenethyl [14,15,16] and most reports stem from *Zyzzya* sponges, though sponges of other genera and even protists are known sources [17]. Tsitsikammamines are characterized by a bispyrroloiminoquinone structure and were first isolated from the South African sponge *Tsitsikamma favus* [18,19], and more recently from Australian *Zyzzya* sp. [10], Antarctic *Latrunculia biformis* [20], and Tongan *Strongylodesma tongaensis* [21]. This structural motif is, however, not restricted to sponges and the related bispyrroloiminoquinone wakayin has been isolated from a Fijian ascidian [22]. Discorhabdins, exhibiting a peculiar spiro-structure [19,23,24], as well as discorhabdin oligomers [25,26,27,28,29] have to date only been reported from marine sponges with the vast majority of studies focusing on *Latrunculia* species [12,13].

Four of the six species analyzed in this study have been the subject of previous chemical investigations. Accordingly, *T. favus* has been reported to produce tribrominated analogues of discorhabdins C and V as well as tsitsikammamines [18,19] and recent investigations by our group have revealed a second chemotype (II) within this species, characterized by the presence of unbranched makaluvamines and damirones [30]. *Tsitsikamma pedunculata* has been reported to be the source of several di- and tribrominated C-series discorhabdins including 3-dihydrodiscorhabdin C and discorhabdin V [19], while *Cyclacanthia bellae* was reported to produce makaluvamines, C-series discorhabdins, and sulfur-containing discorhabdins of the A- and D-series [19]. Antarctic *Latrunculia apicalis* has been the source of discorhabdins C and G (=2-debromo-1,2-dihydro-7,8-dehydrodiscorhabdin C) [31], while the chemistry of the recently described species *Tsitsikamma nguni* and *Tsitsikamma michaeli* [32] is here investigated for the first time.

Most studies on the pyrroloiminoquinone chemistry produced by latrunculid sponges are based on the isolation of compounds and their structural elucidation using NMR techniques [13], but several recent investigations have begun to probe the secondary metabolite reservoir present within these sponges at a broader level using collision-induced dissociation (CID) mass spectrometry methods [20,27,28,30,33,34,35]. Nevertheless, CID spectra of pyrroloiminoquinones are scarce in literature and their dissociation behavior is largely unknown. To gain a deeper insight into the pyrroloiminoquinone chemistry present within latrunculid sponges, we analyzed 43 specimens of the South African sponge species *T. favus* (15 specimens)*, T. nguni* (4)*, T. pedunculata* (10)*, T. michaeli* (9)*, C. bellae* (2), and subantarctic *L. apicalis* (3) using a LC-ESI-MS/MS-based molecular networking approach. Dereplication of the detected ion features was facilitated by molecular formula matching to known pyrroloiminoquinones, spectral comparison to pyrroloiminoquinone isolates and contextualization of characteristic product ions and fragmentation patterns. The analysis revealed distinct species-specific chemical profiles including more than 200 known and unknown putative pyrroloiminoquinones and related compounds.

## 2. Results

**Figure 2:**
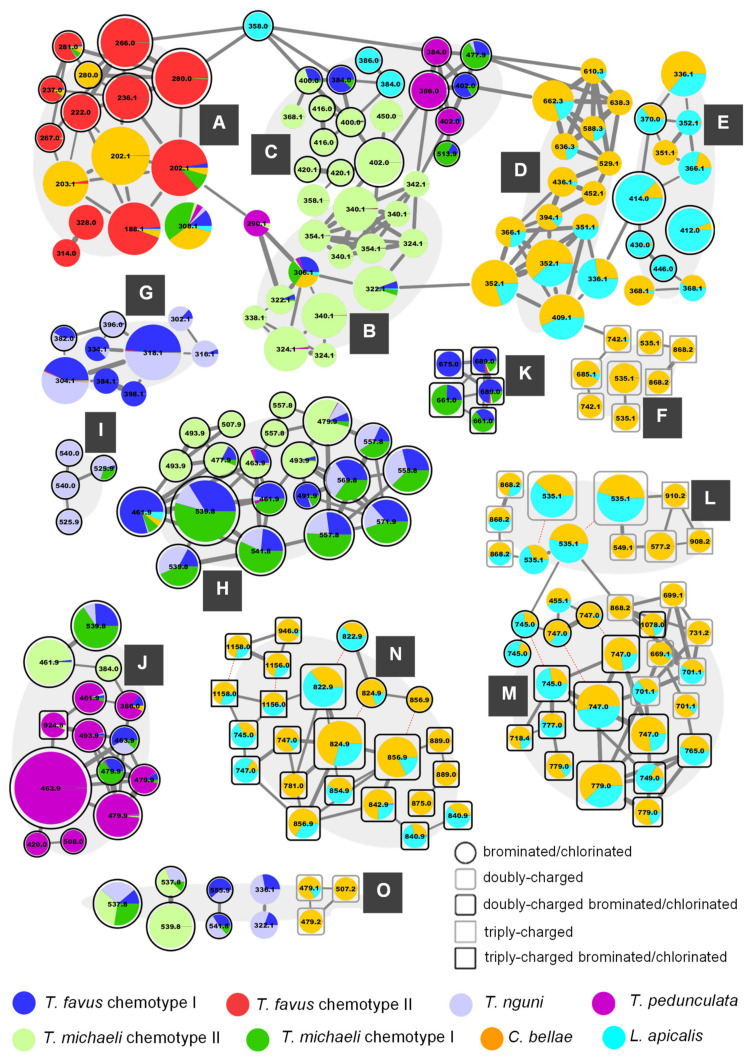
The Molecular network of eight chemically distinct sponge types from six Latrunculiidae species. (**A**) Makaluvamines and damirones; (**B**) Discorhabdin I and hydroxylated derivatives; (**C**) Brominated and hydroxylated Discorhabdin I derivatives. (**D**) Discorhabdins of the D-series; (**E**) Discorhabdins of the A-series; (**F**) Unusual didiscorhabdins and discorhabdin-makaluvamine adducts; (**G**) Tsitsikammamines; (**H**) Di- and tribrominated C-series discorhabdins; (**I**): Putative oxygenated C-series Discorhabdins (**J**) 3-dihydrodiscorhabdins of the C-series; (**K**) ovothiol-substituted C-series discorhabdins; (**L**) ovothiol-substituted A/D-series discorhabdins; (**M**) Disulfide-linked discorhabdin A/D adducts; (**N**) Thioether-linked Discorhabdin A/D adducts; (**O**) Other pyrroloiminoquinones or related compounds. Node contributions correspond to average peak area across all extracts per sponge type. Increasing node size corresponds to greater peak area averaged across all sponge types. Increasing edge width indicates greater spectral similarity. Dashed red lines connect different charge-states of the same feature.

For this study, we accessed an archive of 43 latrunculid sponge specimens collected between 2015 and 2018, predominantly from the southeast coast of South Africa (Agulhas bioregion) and off Bouvet Island in the Southern Atlantic Ocean (Table S1). For the molecular networking analysis, sponge samples were prepared in methanol (MeOH) from frozen sponge material or dried sponge extract (DCM/MeOH; 2/1, *v*/*v*) and subsequent RP-LC-ESI-MS/MS was carried out in positive mode using collision energies of 40 eV. After acquisition, the raw data were converted to mzXML and processed in MZmine2 (ver. 2.34) [36] to create MS^1^-based peak lists with feature-associated MS^2^ spectra. The MS^2^ data ensemble was then deconvoluted into a network, based on spectral similarity using the GNPS feature based molecular networking (FBMN) workflow [37,38]. Prior to this analysis, four molecular networks were created for subsets of the samples (*T. favus* and *T. nguni*, *T. pedunculata*, *T. michaeli* and *C. bellae* and *L. apicalis*; see Figures S2.1–S2.4) confirming that grouping sponge samples into eight different types and averaging across each type for the ‘holistic’ analysis presented in this article was appropriate and did not combine chemically disparate samples. The combined molecular network (Figure 2) shows all clusters as well as the most abundant singleton node, which were annotated as pyrroloiminoquinones or related compounds (Figure S2.5 illustrates the remainder of the network). The nodes of the pyrroloiminoquinone molecular network were organized into 15 groups (A-O) based on molecular network connectivity and structural similarity as per annotation. Importantly, the labels of the nodes were set to correspond to the singly-charged monoisotopic mass of the respective feature to simplify molecular formula identification. This is relevant for multiply-charged and multiply-halogenated features. For multiply halogenated features, the highest abundance isotopomer was selected for MS^2^ analysis, as this provided the highest intensity signal (e.g., ^79^Br^81^Br, ^79^Br_2_^81^Br) and the node label was determined by subtracting two neutron masses from the precursor *m*/*z* value. For multiply charged features, the precursor *m*/*z* was multiplied by the charge (n) followed by subtraction of n-1 proton masses. As a result, the precursor *m*/*z* values in the product ion spectra of these nodes differ from the mass labels presented in Figure 2. Nodes sharing identical labels correspond to separate isomers or, if connected by dashed red lines, to different charge states of the same parent compound.

### 2.1. Group A—Makaluvamines and Damirones

Group A of the molecular network in Figure 2 summarizes features annotated as makaluvamines and damirones and for several of these the annotations were corroborated by spectral matching to reference MS^2^ spectra obtained from isolated compound material (large 280.0 Da node = makaluvamine Q, 281.0 Da node = makaluvone, 267.0 Da node = node = makaluvamine O, right 202.1 Da node = makaluvamine A, 188.1 Da node = makaluvamine I [30], left 202.1 Da node = makaluvamine C (Supplementary Materials S1)). Clear distinctions were observed when comparing the MS^2^ spectra of annotated unbranched makaluvamines and damirones (Figures S3.1–S3.3). Most features in this group were dominated by contributions from *T. favus* chemotype II, with only small contributions from *T. favus* chemotype I samples consistent with previous findings regarding the distinction of *T. favus* chemotypes [30]. Similarly, small contributions to some makaluvamines were made by *T. michaeli* samples from Riy Banks reef (chemotype I) and *L. apicalis*. Furthermore, three features annotated as makaluvamine C (202.1 Da) and damirone C (203.1 Da) as well as an isomer of makaluvamine Q (280.0 Da), were detected almost exclusively in *C. bellae* extracts.

Notable neutral losses for putative unbranched makaluvamines included CHN and CH_3_NO, whereas the fragmentation patterns of damirones were characterized by neutral losses involving CO. Furthermore, several odd-electron neutral losses were observed. For instance, putative *N*-methylated and brominated makaluvamines were found to undergo fragmentation, resulting in product ions matching neutral losses of hydrogen, methyl, and bromine radicals (Figures S3.1–S3.3). Odd-electron product ions from even-electron precursors are unusual when using collision-induced dissociation (CID) methods [39] but analogous neutral losses have been reported in literature from *N*-methylated and brominated precursors [40] and previous studies on makaluvamines also reported odd-electron product ions [41]. This could indicate a pronounced ability of the condensed and conjugated pyrroloiminoquinone core to stabilize a lone electron, and/or a low kinetic barrier between the precursor ions and such odd-electron products.

The feature at 308.1 Da was not connected via a network edge to the rest of the group A features but was nevertheless included due to its annotation as makaluvamine D (=7,8-dihydromakaluvamine M). A reference spectrum of makaluvamine D [41], while being acquired on an alternative instrument type and showing some differences (QToF vs. Ion trap), was found to share a dominant *m*/*z* 201 product ion, matching neutral loss of the side-chain (-•C_7_H_7_O) and supporting the annotation here (Figure S3.4). This feature was detected in all sponge types with major contributions from Riy Banks *T. michaeli* as well as *C. bellae* (very small contribution from *T. favus* chemotype II, hence not resolved in Figure 2). Additionally, two nodes at 314.0 and 328.0 Da were also not connected to the other nodes of group A but were included here due to their putative annotation as iodinated makaluvamine analogues based on matching precursor molecular formulae and neutral losses matching •I and HI (Figure S3.5).

### 2.2. Group B—Discorhabdin I and Hydroxylated Derivatives

The features in group B were annotated as discorhabdin I (306.1 Da) and hydroxylated derivatives of this compound. Importantly, putative discorhabdin I was detected in all sponges except the discorhabdin-deficient *T. favus* chemotype II, possibly reflecting a central role in the biosynthesis of more complex discorhabdins as has been previously suggested for that compound [42]. The nodes of group B are connected to group A via a node at 290.1 Da, which we suspect may represent a discorhabdin decomposition product matching discorhabdin I, lacking one oxygen atom. The other features in this group were annotated as hydroxylated discorhabdin I derivatives and almost exclusively contributed to by Evans Peak *T. michaeli* (proposed as chemotype II) samples with only small contributions from *T. favus* chemotype I, *T. nguni*, Riy Banks *T. michaeli* (chemotype I) and *T. pedunculata* to a few features. Interestingly, gap-filling allowed to predict a low abundance of the putative dihydroxylated discorhabdin I derivative represented by the uppermost 340.1 Da node in the *T. favus* chemotype II extract, indicating that this sponge type is not entirely devoid of discorhabdins.

**Figure 3:**
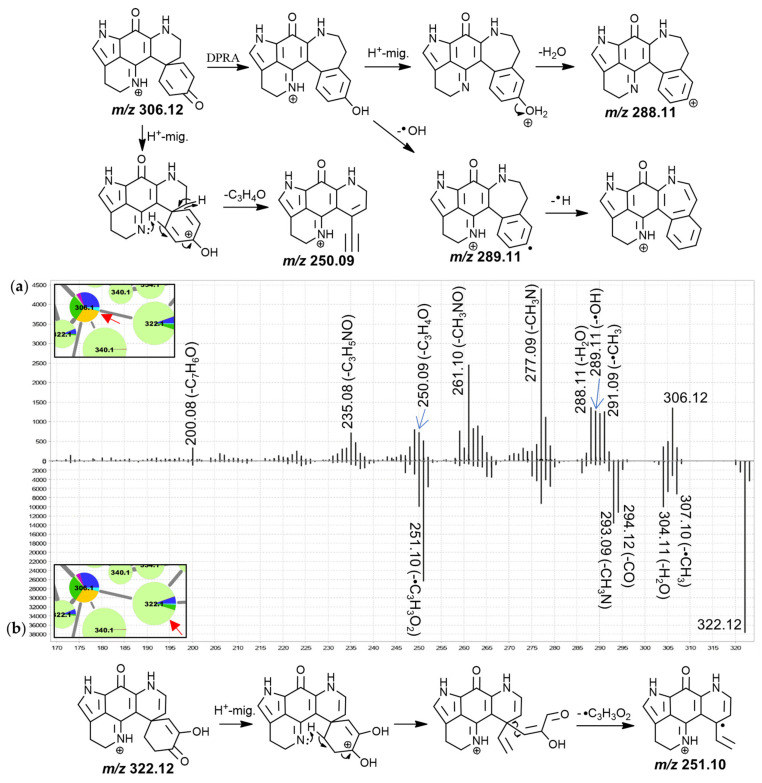
MS^2^ spectra of the 306.1 Da (**a**), (annotated as discorhabdin I) and the rightmost 322.1 Da nodes (**b**), (annotated as an oxygenated discorhabdin I derivative) from group B and proposed key fragmentation mechanisms.

The MS^2^ spectrum of putative discorhabdin I (Figure 3) included product ions at *m*/*z* 291.09 (-•CH_3_), 290.09 (-CH_4_), 289.11 (-•OH), 288.11 (-H_2_O), 277.09 (-CH_3_N), 261.10 (-CH_3_NO) and 250.09 (-C_3_H_4_O). The product ions resulting from radical demethylation and methane loss are unexpected when considering the proposed precursor ion structure and would have to be accompanied by rearrangement. However, corresponding fragments were also observed in the high-resolution spectrum of a makaluvamine I isolate [30] and the matching spectrum for the 188.1 Da node (Figure S3.2), suggesting a dissociation involving atoms of the pyrroloiminoquinone core. This methyl radical loss is subject of ongoing investigation. Furthermore, neutral losses of a hydroxyl radical and water were also unusual and we propose a dienone-phenol rearrangement (DPRA) of the precursor upon collision-induced activation, prior to dissociation, to explain this observation. This would avail a hydroxyl-group for subsequent elimination. Hydroxyl radical losses during CID are seldomly encountered and are usually characteristic of aromatic nitro groups and oximes [40,43]. We speculate that an ability of the pyrroloiminoquinone core to stabilize a lone electron may enable radical hydroxyl elimination after prior dienone-phenol rearrangement, resulting in observation of such a neutral loss.

A CH_3_N neutral loss as observed for the putative discorhabdin I in Figure 3 does not feature prominently in the MS^2^ spectra of makaluvamines and therefore involvement of the condensed phenethyl moiety may be suspected. Contrarily, the CH_3_NO (possibly CHN+H_2_O) neutral loss could involve the pyrroloiminoquinone core, since similar fragments were observed for makaluvamines (Figures S3.1–S3.3). Furthermore, *m*/*z* 249.09, 250.09 and 251.10 product ions were detected. Importantly, the precursor and product ion *m*/*z* values were underestimated by about 10 to 40 ppm in the LC-MS/MS experiments of this analysis as determined from interrogating precursor and product ion *m*/*z* values of compounds matched to isolated material. When limiting the molecular formula search for the precursor to at least include the atoms of the pyrroloiminoquinone core as well as carbon, hydrogen, nitrogen, and oxygen, only the formula of the annotated discorhabdin I was within a −10–40 ppm window (−13.89 ppm). Searching a molecular formula for the *m*/*z* 250.09 fragment with the upper limitation of the annotated precursor formula revealed two possible candidate formulae, C_15_H_10_N_2_O_2_^+^ (−32 ppm) and C_17_H_14_O_2_ (−38 ppm). These formulae correspond to neutral losses of C_3_H_4_O and CN_3_ from the precursor, respectively. Since the nitrogen atoms can reasonably be expected to reside in the pyrroloiminoquinone core and no matching neutral losses were seen for makaluvamines, the latter neutral loss is exceedingly unlikely and the product ion formula was therefore annotated as C_15_H_10_N_2_O_2_^+^. The formulae of the neighboring product ions at *m*/*z* 249.09 and 251.10 were matched to C_15_H_9_N_2_O_2_^+•^ and C_15_H_11_N_2_O_2_^+•^, respectively.

The corresponding neutral losses are suspected to be characteristic of discorhabdins and to involve the dienone-moiety since this represents the only region of the proposed precursor structure where the respective atoms can be found in coherent positions. This moiety represents a distinguishing structural alteration compared to other pyrroloiminoquinones, such as makaluvamines and tsitsikammamines, for which no such product ions were observed. We propose the underlying CID mechanism to involve initial proton migration according to the mobile proton hypothesis [44] from N-18 to O-3 followed by remote hydrogen rearrangement [39] and a β-hydrogen removal-mediated dissociation involving N-18 [39]. The adjacent odd-electron *m*/*z* 249.09 and 251.10 product ions could be formed via competing reactions involving homolytic C-C cleavage instead of remote hydrogen rearrangement. Precursor ions with *tert*-butyl groups have been shown to provide CID product ions matching methyl radical neutral loss [43] indicating that C-C homolytic cleavage, while unusual, is possible under special circumstances.

The spectrum of the 322.1 Da node (Figure 3b) exhibited similar neutral losses but a prominent *m*/*z* 251.10 product ion was observed. We propose that prominence of this fragment may be indicative of a 7,8-dehydro bond since this would inhibit a remote hydrogen rearrangement, as was proposed for discorhabdin I, and stabilize a putative tertiary radical product ion. The spectra of the remaining features in this group were characterized by neutral losses of water and CO and most spectra exhibited unusual odd-electron product ions at *m*/*z* 251.10 (C_15_H_11_N_2_O_2_^+•^, −23 ppm).

### 2.3. Group C—Monobrominated and Hydroxylated C- and V-Series Discorhabdins

**Figure 4:**
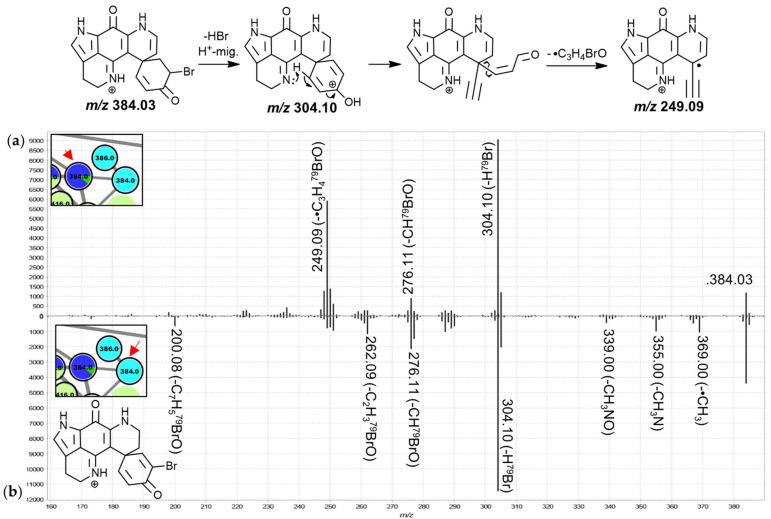
MS^2^ spectra of the leftmost 384.0 Da (**a**) and the central 384.0 (**b**) node from group C (putative C-series discorhabdins) and proposed key fragmentation mechanisms.

The features of group C showed distinctive sponge type-specific contribution profiles and close spectral similarity to the features in group B partly due to neutral losses of CO, H_2_O as well as *m*/*z* 251.10–249.09 product ions. They were mostly annotated as monobrominated and monobrominated-hydroxylated discorhabdins with exceptions being the nodes at 368.1 and 450.0 Da which were annotated as thiomethylated and iodinated discorhabdin analogues (Figure S3.6), respectively, as well as the nodes at 513.9 and 477.9 Da, which represented dibrominated precursor ions. This subcluster was connected to group A via a node at 358.0 Da matching a molecular formula of C_16_H_13_BrN_3_O_2_ and producing a *m*/*z* 250.09 fragment, suggesting that this feature may represent a discorhabdin derivative or decomposition product. We suspect that the features of the left portion of group C, with comparably low residual precursor ion intensities in their product ion spectra, represent C-series discorhabdin derivatives (Figure 4), whereas those of the portion on the right likely represent V-series discorhabdins exhibiting N(18)-C(2) ring closure and product ion spectra with high residual precursor ion intensities (Figure S3.7). This may be a consequence of increased structural stability and/or higher kinetic barriers for CID reactions of the N(18)-C(2) condensed V-series discorhabdins opposed to C-series discorhabdins. Importantly, three features annotated as V-series discorhabdins were contributed to primarily by *T. pedunculata* samples (384.0, 386.0 and 402.0 Da), while the neighboring features at 402.0, 477.9 and 513.9 Da were prevalent in *T. favus* and *T. michaeli* chemotypes I and *T. nguni*. The majority of putative discorhabdins in group C were detected only in *T. michaeli* chemotype II, while one isomer matching a monobrominated C-series discorhabdin (384.0) was detected in *T. favus* chemotype I, *T. michaeli* chemotype I and *T. nguni* whereas another isomer was detected in *L. apicalis*.

The MS^2^ spectra of these two leftmost 384.0 Da nodes (Figure 4) were found to exhibit similar product ion distributions with major neutral losses corresponding to HBr (*m*/*z* 304.10) and CHBrO (*m*/*z* 276.11). However, the precursor predominantly detected in chemotype I of both, *T. favus* and *T. michaeli*, showed a much more pronounced *m*/*z* 249.09 fragment, whereas other minor neutral losses were observed for the isomer detected in *L. apicalis* such as •CH_3_, CH_3_N, C_2_H_3_BrO and C_7_H_5_BrO. The latter spectrum resembled that of the feature annotated as discorhabdin I with relatively low abundance product ion signals between *m*/*z* 249 and 251, leading us to propose that the feature at hand corresponds to the known discorhabdin E, while the other node was annotated as a regioisomer of discorhabdin G.

### 2.4. Group D—D-series Discorhabdins

The features in group D were mostly annotated as D-series discorhabdins. The contributions to most features were dominated by *C. bellae* samples with significant contributions from the *L. apicalis* samples and no contributions from any of the other sponge types. The annotated structures include the known compounds discorhabdin D (336.1 Da), 1-aminodiscorhabdin D (351.1 Da), discorhabdin L (352.1 Da), 1-acetyldiscorhabdin L (394.1 Da), discorhabdin N (409.1 Da), as well as one C(1)-methoxylated A-series discorhabdin (366.1 Da). The nodes at 436.1 Da and 452.1 Da were annotated as C(1)-substituted discorhabdin D analogues, while the remaining nodes at 529.1, 588.3, 610.3, 636.3, 638.3, and 662.3 Da likely represent similar D-series discorhabdin adducts.

**Figure 5:**
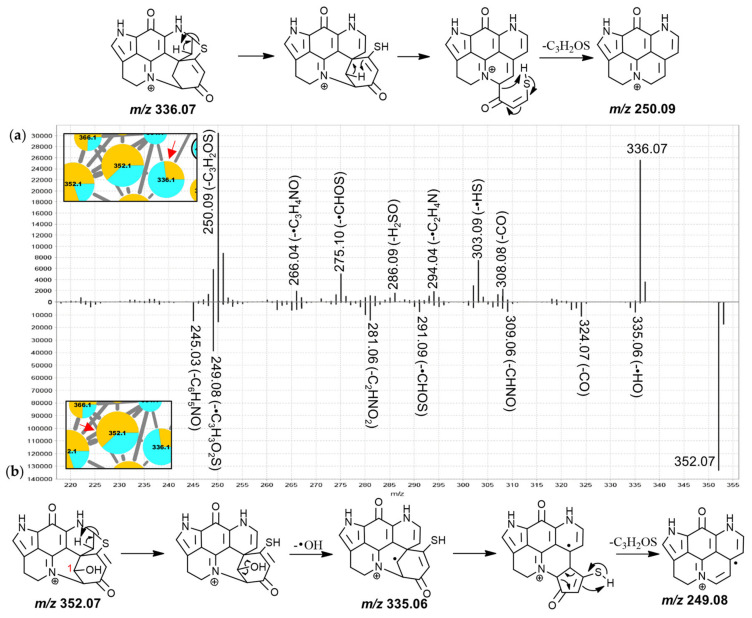
MS^2^ spectra of the 336.1 Da node (**a**), (annotated as discorhabdin D) and the right 352.1 Da node (**b**), (annotated as discorhabdin L) from group D and proposed key fragmentation mechanisms.

Generally, the MS^2^ spectra of the nodes in this sub-cluster showed relatively high residual precursor ion intensities and abundant *m*/*z* 249.08/250.09 product ions, distinguishing them from group E (mostly annotated as A-series discorhabdins) where more abundant fragments were observed in the *m*/*z* range between 249 and the precursor *m*/*z* value. These product ions correspond to the same ion formulae as the 249.09 and 251.10 discussed within group B with the *m*/*z* difference being attributed to mass imprecision in the experimental series. A *m*/*z* 249 fragment has been reported in literature for the product ion spectra of several putative A- and D-series discorhabdins using FT-HRESIMS (detailed experimental parameters were not provided) including discorhabdin D and discorhabdin H, which both also gave *m*/*z* 303 fragments [34]. For the 336.1 Da node annotated as discorhabdin D in group D the most abundant MS^2^ signals were found at the residual precursor ion mass, as well as at *m*/*z* 250.09. Our proposed fragmentation mechanism towards the *m*/*z* 250.09 product ion involves three remote-hydrogen rearrangements leading to a pentacyclic product ion structure (Figure 5a).

For discorhabdin L, key MS^2^ fragments have been reported at *m*/*z* 335, 324, 281 and 250 [45]. Equivalent product ions were observed for the annotated discorhabdin L (352.1 Da, both isobaric nodes exhibited identical MS^2^ spectra and may represent stereoisomers) in the analysis at hand (Figure 5b). However, a *m*/*z* 249.08 fragment was detected more abundantly than a *m*/*z* 250.09 signal. This could be a consequence of differing instrumental parameters (e.g., collision energy, experimental time frame) and instrument types (QToF vs. Ion trap). Furthermore, 1-acetyl-discorhabdin L, recently isolated from *L. biformis* [33], was annotated for the 394.1 Da node. This compound was reported to provide a distinct *m*/*z* 352 product ion [33] that was also observed here (Figure S3.8) as well as for the remaining higher mass features in this group.

### 2.5. Group E—A-Series Discorhabdins

The nodes in group E were mostly annotated as A-series discorhabdins and most features, especially those representing brominated analogues, were predominantly contributed to by *L. apicalis* samples. The MS^2^ spectra of the compounds in group E were characterized by similar neutral losses as those found for the features in group D, albeit with higher intensity product ions in the window between the precursor *m*/*z* value and *m*/*z* 251.

**Figure 6:**
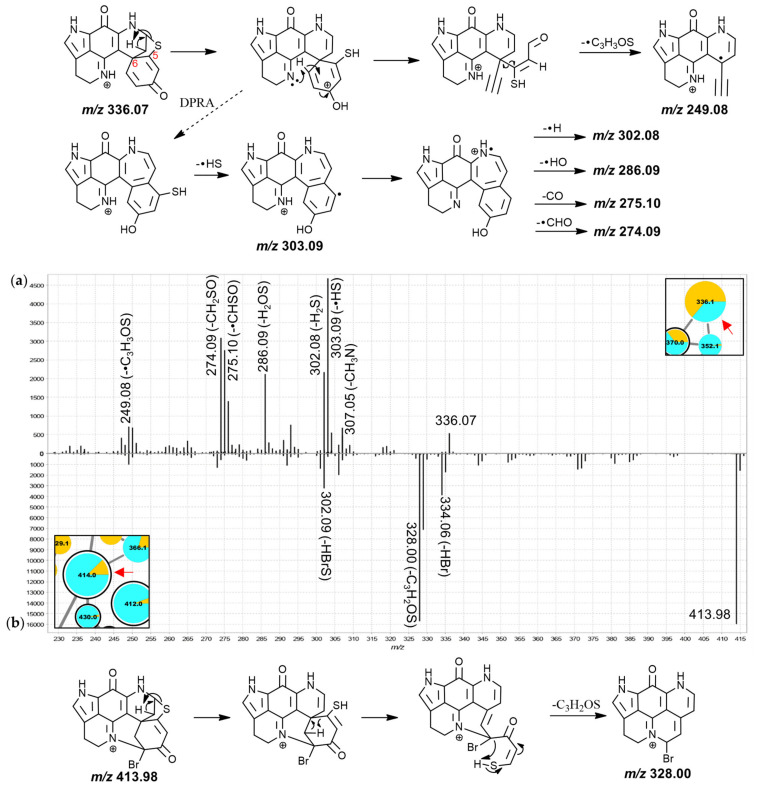
MS^2^ spectra of the 336.1 Da node (**a**), (annotated as discorhabdin G*) and the 414.0 Da node (**b**), (annotated as 2-bromodiscorhabdin D) from group E and proposed key fragmentation mechanisms.

Inspection of the MS^2^ spectra of the 336.1 Da and the 414.0 Da nodes led to their annotation as the known discorhabdins G* and 2-bromodiscorhabdin D, respectively. For the 336.1 Da compound (Figure 6a), particularly abundant product ions were found at *m*/*z* 303.09, 286.09, and 274.09, possibly produced along the same fragmentation path and corresponding to neutral losses of •SH, H_2_SO and CH_2_SO, respectively. The odd-electron product ion at *m*/*z* 249.08 corresponded to neutral loss of •C_3_H_3_SO from the precursor ion. A putative fragmentation mechanism towards this product ion (C_15_H_9_N_2_O_2_^+•^, underestimated by −41 ppm; precursor at *m*/*z* 336.07 for C_18_H_14_N_3_O_2_S^+^ underestimated by −32 ppm) is proposed in Figure 6a, involving remote-hydrogen rearrangement, proton migration, β-hydrogen removal and homolytic cleavage. An odd-electron product ion corresponding to neutral loss of •SH was detected at *m*/*z* 303.09 and it is possible that the product ions at *m*/*z* 302.08 (-H_2_S), 286.09 (-H_2_SO), 275.10 (-•CHSO) and 274.09 (-CH_2_SO) originate along the same fragmentation channel as this *m*/*z* 303.09 product ion, hypothetically involving a dienone-phenol rearrangement as was proposed for putative discorhabdin I (Figure 3a).

The MS^2^ spectrum of the 414.0 Da node showed a high-abundance fragment derived from neutral loss of C_3_H_2_OS at *m*/*z* 328.00 (matches *m*/*z* 250.09 fragment with bromine instead of hydrogen) prompting an annotation as a hexacyclic D-series discorhabdin for the precursor compound (Figure 6b). The molecular formula of this compound was identical to the known pentacyclic discorhabdin B, which has been reported to exhibit major CID-fragments at *m*/*z* 334 and 302 using an ion trap instrument [45]. A *m*/*z* 328.00 fragment was, however, not reported supporting the annotation here of a different isomer, namely 2-bromodiscorhabdin D, a pyrroloiminoquinone recently isolated for the first time from *L. biformis* [33]. The central 366.1 Da node was annotated as a new 2-methoxydiscorhabdin D while the 412.0 Da node was annotated as the known discorhabdin Q (Figure S3.9).

### 2.6. Group F—New Didiscorhabdins and Discorhabdin-Makaluvamine Adducts

The sub-cluster denoted as group F in the molecular network (Figure 2) is derived from six doubly-charged and three triply-charged precursor ions. All nine compounds were found predominantly in *C. bellae* extracts with trace amounts of only the 685.2 Da and one 742.2 Da node being detected in *L. apicalis*. Inspection of the MS^2^ spectra of the corresponding features led to their annotation as dimeric and trimeric pyrroloiminoquinone adducts of either two A- or D-series discorhabdins, one A-/D-series discorhabdin and one makaluvamine, or two A-/D-series discorhabdins and one makaluvamine. The mode of linkage of the respective monomers remains unclear and may correspond to thioether bridges as exemplified in the known discorhabdin B dimers [26,27] or to C-N linkage as reported for some discorhabdin di- and trimers [28,29].

**Figure 7:**
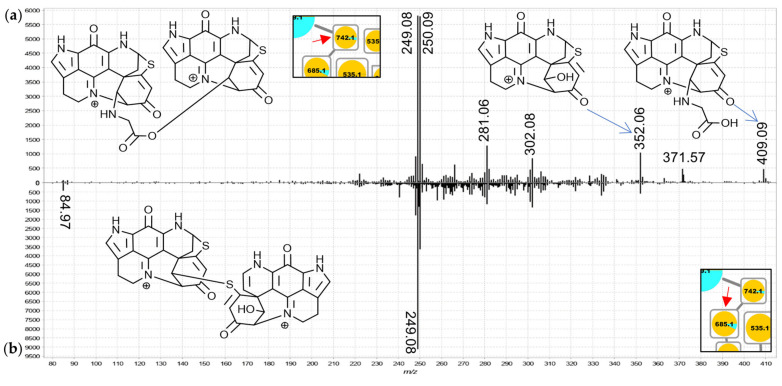
MS^2^ spectra of the 742.2 Da (**a**), (annotated as a glycine-linked discorhabdin dimer) and the 685.2 Da node (**b**), (annotated as a discorhabdin dimer) from group F (extended spectra can be found in Figure S3.10).

**Figure 8:**
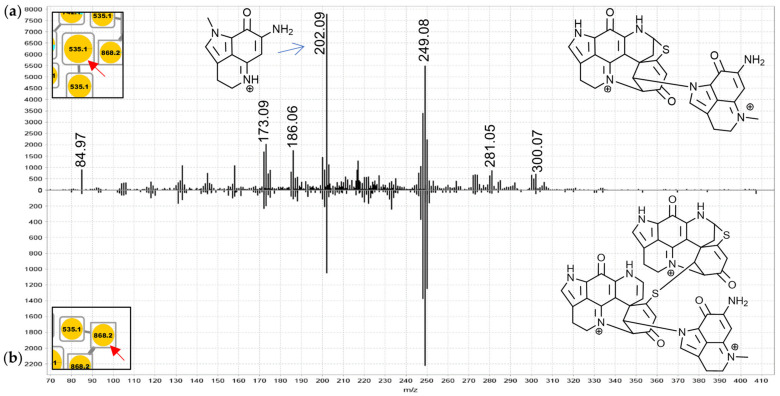
MS^2^ spectra of the larger 535.2 Da (**a**), (annotated as a discorhabdin-makaluvamine dimer) and the upper 868.2 Da node (**b**), (annotated as a discorhabdin-discorhabdin-makaluvamine trimer) from group F (extended spectra can be found in Figure S3.11).

For the 742.2 Da nodes, linkage through a bridging glycine is suspected because the MS^2^ spectra exhibited distinct product ions at *m*/*z* 409.09 and 352.07 matching discorhabdin N and discorhabdin L, respectively (Figure 7a). This observation leads to a proposed structure comprised of two discorhabdin moieties linked via glycine. The MS^2^ spectrum of the 685.2 Da node (Figure 7b) is similar, albeit without a *m*/*z* 409.09 product ion. Therefore, we propose linkage of two discorhabdin monomers via a sulfur-bridge, in the same manner as reported for the known discorhabdin B-dimer [26,27]. Finally, the MS^2^ spectra of the larger 535.2 Da node (Figure 8a) and the upper 868.2 Da node (Figure 8b) contained evidence for discorhabdin (*m*/*z* 302.08, 249.08) and makaluvamine A or C (*m*/*z* 202.09 and 186.06 compare Figures S1.3 and S3.1) product ion prompting annotations as discorhabdin-makaluvamine oligomers.

### 2.7. Group G—Tsitsikammamines

The features in group G, annotated as tsitsikammamines, were particularly abundant in *T. favus* chemotype I and *T. nguni*. Low relative levels of the 304.1 and 318.1 Da features were detected in *T. favus* chemotype II and *C. bellae* samples. The annotations in this group include tsitsikammamine A (304.1 Da) and B (318.1 Da), their 16,17-dehydro analogues (302.1 and 316.1 Da), a hydroxylated tsitsikammamine B analogue (334.1 Da), as well as brominated (382.0 and 396.0 Da) and putative sulfate derivatives (384.1 and 398.1 Da). The MS^2^ spectra of the majority of features were found to exhibit two distinct types of fragments, a product ion at *m*/*z* 211.04 and at *m*/*z* 159.05 or 145.03 depending on whether *N*-methylation is present in the precursor or not (e.g., Figures S3.12 and S3.13). Only the putative 16,17-dehydro analogues did not exhibit such distinctive fragments, but rather showed neutral losses of CO and C_2_HNO (Figure S3.12b).

### 2.8. Group H—Di- and Tribrominated C-Series Discorhabdins

**Figure 9:**
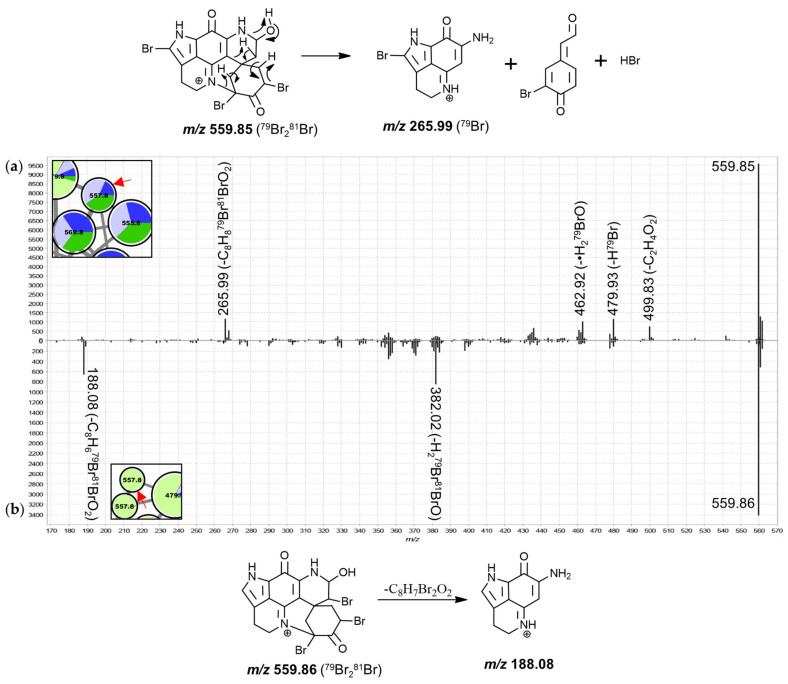
MS^2^ spectra of the rightmost 557.8 Da node (**a**) and the uppermost 557.8 Da node (**b**) from group H and proposed key fragmentation mechanism.

The features in group H were annotated as di- and tribrominated C-series discorhabdins, supported by previous reports of such discorhabdins from *T. favus* [18,19]. The product ion spectra of these features were dominated by fragments resulting from debrominations (Figure S3.14) and most features, especially those corresponding to tribrominated precursors, were primarily contributed to by *T. favus* and *T. michaeli* chemotypes I, as well as *T. nguni*. Interestingly, one feature, annotated as a dibrominated C-series discorhabdin, was contributed to by *C. bellae* and *L. apicalis* indicating that dibromination of discorhabdin monomers does occur in these sponges. *T. michaeli* chemotype II samples dominated contributions to the nodes annotated as dibrominated hydroxylated discorhabdins. Furthermore, two tribrominated features (557.8 Da) were exclusively detected in *T. michaeli* chemotype II with two isobaric features occurring in *T. favus* and *T. michaeli* chemotypes I and *T. nguni*. Noteworthy, the 557.8 Da features found in *T. michaeli* chemotype II compounds showed product ions at *m*/*z* 188.08 matching makaluvamine I, whereas the isobaric features present in *T. favus* and *T. michaeli* chemotypes I as well as *T. nguni* showed product ions at *m*/*z* 265.99 (Figure 9). This suggests that for the two features in *T. michaeli* chemotype II, tribromination may occur at the spiro-moiety without involving C-14.

### 2.9. Group I—Oxygenated C-Series Discorhabdins

The four features in group I were mainly detected in *T. nguni* extracts. These nodes comprise the only putative pyrroloiminoquinone cluster dominated by contributions from *T. nguni* samples and may be of significance for the chemotaxonomic identification of this species but could also be an artifact of prolonged sample storage at −20 °C. Three of these nodes were exclusively detected in *T. nguni* samples, while one node at 525.9 Da was also detected in some *T. favus* chemotype I samples from both collection sites (see Figure S2.1). These compounds potentially represent new pyrroloiminoquinones, yet the MS^2^ spectra do not provide much information (Figure S3.15). However, their annotation as discorhabdins is supported by the presence of *m*/*z* 329.01/331.01 brominated fragments, which may correspond to the same brominated fragments observed for the major node in group I (Figure S3.14b). If this is correct, then bromination of the pyrroloiminoquinone core at C-14 may be indicated.

### 2.10. Group J—3-dihydrodiscorhabdins of the C-Series

**Figure 10:**
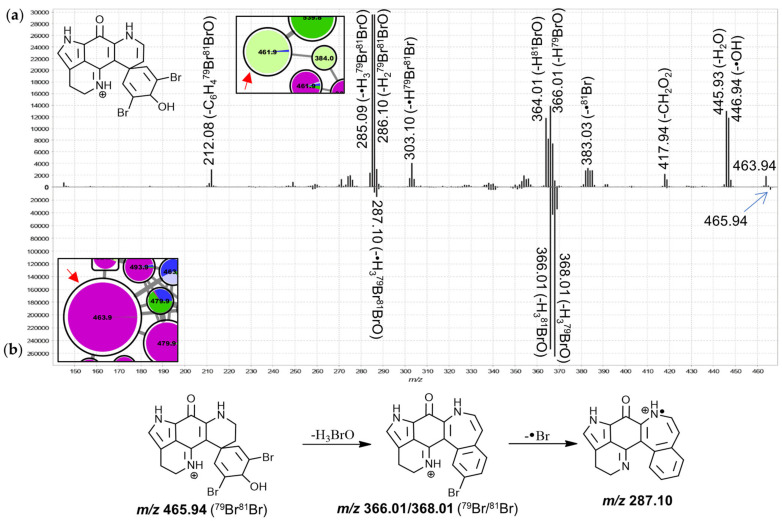
MS^2^ spectra of the large 461.9 Da node (**a**), ^79^Br^81^Br precursor, (annotated as 7,8-dehydro-3-dihydrodiscorhabdin C) and the 463.9 Da node (**b**), ^79^Br^81^Br precursor, (annotated as 3-dihydrodiscorhabdin C) from group J.

The features in group J were annotated as 3-dihydrodiscorhabdin C derivatives, due to spectral matching of the large 463.9 Da node to isolated 3-dihydrodiscorhabdin C reference material (Supplementary Materials S1). The MS^2^ spectra of these nodes are highly distinct from those of features annotated as C-3 carbonyl discorhabdins and show mainly product ions derived from neutral losses of H_2_O, HBr and/or combinations thereof while neutral losses involving carbon atoms were much less pronounced (Figure 10). 3-Dihydrodiscorhabdin C showed major neutral losses of H_3_BrO, which in context of the precursor structure necessitates rearrangement (Figure 10b). Most features in this group were dominated by contributions from *T. pedunculata* while some features were most abundantly contributed to by *T. michaeli* chemotype II or *T. favus* and *T. michaeli* chemotypes I as well as *T. nguni*. Of these, the node at 539.8 Da was identified as 14-bromo-3-dihydro-7,8-dehydrodiscorhabdin C through spectral matching to the characterized reference material [30] and the neighboring node at 461.9 Da likely represents 3-dihydro-7,8-dehydrodiscorhabdin C.

### 2.11. Group K and L—Ovothiol-Substituted C-Series and A/D-series Discorhabdins

**Figure 11:**
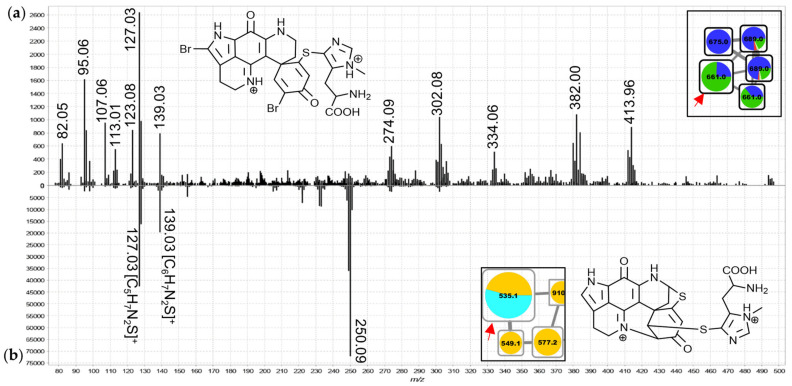
MS^2^ spectra of the 661.9 Da node from group K (**a**), (doubly-charged, ^79^Br^81^Br precursor) and the larger 535.1 Da node from group L (**b**), doubly-charged, annotated as discorhabdin H) from group L (extended spectra can be found in Figure S3.17).

The features in groups K and L were annotated as ovothiol-substituted discorhabdins based on distinctive low *m*/*z* product ions (Figure 11, e.g., *m*/*z* 127.03; new derivatives of C-series discorhabdins in group K and A/D-series discorhabdins in group L). These were initially noticed in the MS^2^ spectrum of the node in group L annotated as the doubly-charged precursor of discorhabdin H (a known secondary metabolite of *C. bellae*; Figure 11b) based on molecular formula matching and chemotaxonomic context. The singly-charged precursor ions of putative ovothiol-substituted discorhabdins showed *m*/*z* 202.06 product ions matching simple cleavage of the side chain (Figure S3.16b). Small contributions to two features in group K were made by *T. favus* chemotype II, further suggesting that this chemotype is not entirely devoid of discorhabdin biosynthetic capacity.

### 2.12. Group M—Disulfide-Linked Discorhabdin A/D Adducts

**Figure 12:**
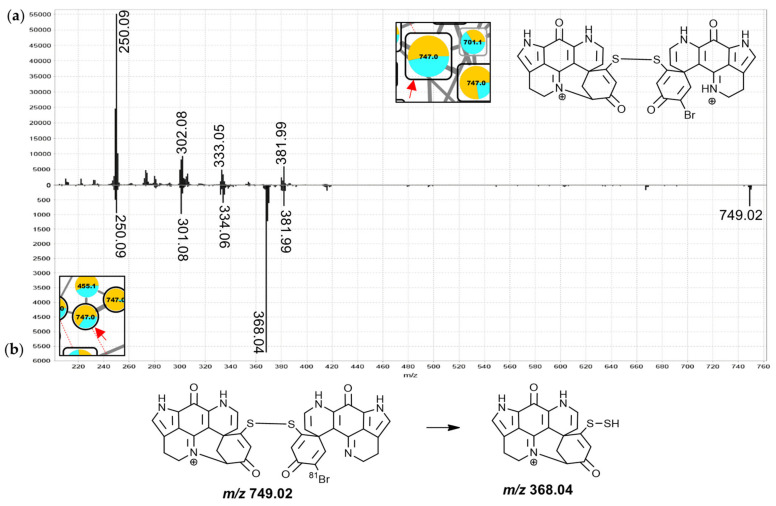
MS^2^ spectra of the central pair of the doubly-charged 747.0 Da node (**a**) and the singly-charged 747.0 Da node (**b**) from group M annotated as a discorhabdin D-discorhabdin B dimer linked through a disulfide-bridge.

Group M in the molecular network is comprised of 23 nodes annotated as discorhabdin dimers linked by a disulfide bridge. These include four nodes of singly-charged ions, three of which were found to correspond to alternative charge-states of compounds also represented by nodes of doubly-charged ions (Figure 2, connected by dashed red lines). Disulfide-linked discorhabdins are exemplified in the known discorhabdin W [25] and the annotations as analogous compounds here were based on the observation of a *m*/*z* 368.04 fragment in the MS^2^ spectra of the nodes of the singly-charged ions at 745.0 and 747.0 Da. The *m*/*z* 250.09 fragments in the MS^2^ spectra of the doubly- and singly-charged precursors at 747.0 Da support their annotation as discorhabdins (Figure 12), while the *m*/*z* 368.04 for the singly-charged precursor (Figure 12b) suggested disulfide-linkage between the discorhabdin monomers, due to the fact that persulfides (R-S-SH) are known CID fragments of disulfide bonds [46,47]. In comparison to the similar mass compounds of group N, the MS^2^ spectra of the nodes of doubly-charged precursors in this group showed particularly abundant *m*/*z* 249.08/250.09 fragments with only low intensity at higher *m*/*z* values and most of the features were contributed to primarily by *C. bellae* samples.

### 2.13. Group N—Thioether-Linked Discorhabdin A/D Adducts

Group N comprises 24 nodes matching mono- and dibrominated compounds, some of which correspond to different charge states of the same precursor compound (Figure 2, connected by dashed red lines). The structures annotated to the nodes in this group are those of halogenated, thioether-linked A-/D- series discorhabdin dimers and trimers, possibly analogous in connectivity to the known discorhabdin B dimer [26,27]. As for the structures annotated in group F, the mode of linkage for the structures annotated in this group could not be conclusively inferred from the experimental data and could therefore include C-N linked discorhabdin dimers similar to pyrroloiminoquinones recently reported [28,29]. Specifically the doubly-charged precursors exhibited fragmentation patterns reminiscent of other putative A- and D-series discorhabdins (Figure S3.18; e.g., *m*/*z* 300.07, 249.09).

### 2.14. Group O—Other Pyrroloiminoquinones or Related Compounds

Group O summarizes all small pyrroloiminoquinone clusters and includes the largest singleton node of the molecular networking analysis at 537.8 Da. The latter feature was detected in *T. favus* chemotype I, *T. nguni* and both chemotypes of *T. michaeli* and annotated as 14-bromo-7,8-dehydrodiscorhabdin C (Figure S3.19a). The neighboring pair of nodes at 537.8 and 539.8 Da was predominantly detected in *T. michaeli* chemotype II samples and they were annotated as new 16,17-dehydro derivatives of 14-bromo-3-dihydrodiscorhabdin C (Figure S3.19b; Figure S3.20a). Furthermore, the nodes at 559.9 and 541.8 Da (Figure S3.20b) are likely to represent unprecedented tribrominated makaluvamine D derivatives, based on a CID behavior similar to what was observed for makaluvamine D (Figure S3.20b vs. group A, Figure S3.3; major neutral loss •C_7_H_5_Br_2_O vs. •C_7_H_7_O). The nodes at 322.1 and 336.1 Da matched the molecular formulae of putative zyzzyanones and showed product ion spectra, exhibiting a dominant ammonia neutral loss, and were unlike any of the spectra of the putative pyrroloiminoquinones discussed above (Figure S3.21). The remaining cluster most likely represents minor A-/D-series discorhabdins since their product ion spectra showed characteristic fragments in the *m*/*z* 249-251 range (Figure S3.22).

## 3. Discussion

The comparative molecular networking analysis of six latrunculid species *T. favus*, *T. nguni*, *T. pedunculata*, *T. michaeli*, *C. bellae* and *L. apicalis* has revealed species-specific pyrroloiminoquinone profiles and provided a comprehensive and informative overview of the relationship between their respective secondary metabolite reservoirs. Despite the structures for most annotated features being putative due to a lack of verified reference material, chemotaxonomic context, molecular masses and fragmentation behavior allowed to identify candidate structures and ring-fence the pyrroloiminoquinone classes represented within the molecular network. For *T. favus*, most specimens (Evans Peak and Bell Buoy) had chemotype I profiles, characterized by the abundance of tsitsikammamines (group G in Figure 2) and brominated discorhabdins of the C- and V-series (group C and group H). As in previous reports [30], *T. favus* chemotype II, characterized by the almost exclusive presence of unbranched makaluvamines, including halogenated derivatives such as makaluvamine Q, was only encountered at Evans Peak reef. The new species, *T. nguni* had an almost identical pyrroloiminoquinone profile to *T. favus* chemotype I, albeit with a slightly higher average ratio of tsitsikammamine/discorhabdin abundance.

This study has provided evidence of a second *Tsitsikamma* species that exhibits two chemotypes; the new species, *T. michaeli*, exhibited regiospecific chemotypes with specimens from Evans Peak and Riy Banks having significantly different pyrroloiminoquinone profiles. At Evans Peak, *T. michaeli* chemotype II sponges contained a variety of compounds annotated as hydroxylated and brominated C-series discorhabdins that were mostly absent in Riy Banks (chemotype I) specimens. *T. michaeli* chemotype I extracts were dominated by the same tribrominated C-series discorhabdins detected in *T. favus* chemotype I, but without tsitsikammamines. The closely related *T. pedunculata* produced a distinct suite of compounds mainly annotated as mono- and dibrominated 3-dihydrodiscorhabdins of the C-series and predominantly 3-dihydrodiscorhabdin C. While a previous investigation reported the isolation of tribrominated C-series discorhabdins from this species [19], we speculate that the analyzed specimens may have included morphologically similar *T. michaeli* chemotype I sponges [32] shown here to produce such tribrominated discorhabdins.

As expected, the chemical profiles of *C. bellae* and *L. apicalis* sponges were distinct from the *Tsitsikamma* sponges. These two species produce remarkably similar pyrroloiminoquinone chemistry despite their more distant taxonomic relationship and occurrence > 1000 km apart and at different depths (12 m vs. 400 m). Both species contained many compounds annotated as sulfur-bridged A- and D-series discorhabdins, including dimers, trimers and adducts thereof. Nevertheless, both species were also well distinguishable by the relative abundance and presence of some putative pyrroloiminoquinones. For instance, *C. bellae* extracts contained comparatively high levels of features annotated as makaluvamine C and damirone C, as well as minor traces of tsitsikammamines, all of which were not detected in *L. apicalis* extracts. Furthermore, the *C. bellae* extracts were found to contain relatively higher levels of most pyrroloiminoquinone dimers and adducts, whereas especially two brominated, sulfur-bridged discorhabdin monomers, annotated as discorhabdin Q (412.0 Da, group E) and 2-bromodiscorhabdin D (414.0 Da, group E), were prevalent in *L. apicalis*. Especially the pyrroloiminoquinone reservoir of *L. apicalis* is reminiscent of Antarctic *L. biformis* [20,27,28,33] rather than previous reports from Antarctic *L. apicalis* [31]. The similarities between the secondary metabolite profiles of these two species points to a conserved pyrroloiminoquinone biosynthetic pathway that we propose may be associated with conserved microbial symbionts [48].

**Figure 13:**
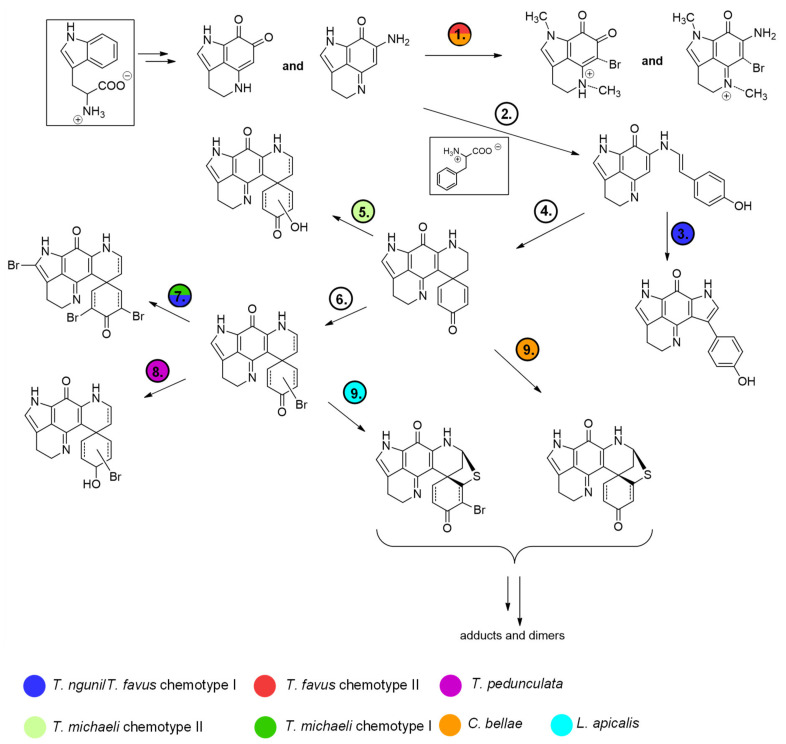
Proposed biosynthetic sequence illustrating the variability of pyrroloiminoquinone production in latrunculid sponges. Reactions that appear to be particularly prevalent in a sponge type according to annotations in the molecular network are indicated by color. 1. *N*-methylation and bromination of makaluvamines and damirones, 2. Formation of branched makaluvamines from unbranched makaluvamines under reaction with phenylalanine, 3. Condensation to tsitsikammamines, 4. Condensation to discorhabdins, 5. Hydroxylation, 6. Bromination, 7. Further bromination, 8. C-3 reduction, 9. Sulfur-insertion.

Interestingly, the features annotated as makaluvamine I (188.1 Da, group A), makaluvamine D (308.1 Da, group A) and discorhabdin I (306.1 Da, group B), all considered central intermediates in the biosynthesis of more complex discorhabdins (Figure 13), were found in all discorhabdin-producing sponges (Figure 2). Putative substituted derivatives of discorhabdin I (e.g., 322.1, 324.1, 384.0 and 386.0 Da present in group B and C) exhibited species-specific distributions. Also noteworthy is the observation that distinct nodes annotated as monobrominated discorhabdins (384.0 or 386.0 Da group C) were each dominant in chemotype I of *T. favus* and *T. michaeli*, *L. apicalis* and *T. pedunculata*. In contrast, none of these putative monobrominated discorhabdins were detected in *T. michaeli* chemotype II; instead many compounds annotated as hydroxylated and monobrominated hydroxylated discorhabdins were found in these sponges. This may infer that biosynthesis in the analyzed discorhabdin-producing sponges concurs up to the production of discorhabdin I and then diverges by derivatization reactions with species-specific rates (Figure 13).

Lastly, interrogation of numerous MS^2^ spectra has revealed that each pyrroloiminoquinone class produces distinct product ions and fragmentation patterns, with ions between *m*/*z* 249–251 deemed characteristic of discorhabdin structures. The occurrence of these unusual odd-electron product ions found for many pyrroloiminoquinones warrants further investigation and mass spectrometry analyses of more discorhabdin isolates may allow us to verify and unravel deeper precursor-fragment relationships. The proposed fragmentation mechanisms presented here provide a basis for future interpretations in CID theory of pyrroloiminoquinones and a starting point for quantum chemical calculations.

## 4. Materials and Methods

### 4.1. Collection of Biological Material

Collection metadata for sample collection are provided in Table S1. Specimens were kept on ice after collection and stored at −20 °C upon arrival in the laboratory. Species identification of specimens was achieved by microscopic spicule analysis and confirmed by 28S rRNA gene sequence for selected sponges as described previously [32]. Genbank accession numbers are provided in Table S1. Collection permits were acquired prior to collections from the Department Environmental Affairs (DEA) and Department Agriculture, Forestry and Fisheries (DAFF) now the Department of Environment, Forestry and Fisheries (DEFF) Research under permit numbers: 2015: RES2015/16 and RES2015/21; 2016:RES2016/11; 2017:RES2017/43; 2018: RES2018/44.

### 4.2. Sample Preparation and Data Acquisition

Chemical extracts from *T. pedunculata* specimens TIC2015-214, TIC2015-216, TIC2015-217, TIC2015-218, TIC2015-219 and *T. michaeli* sponges TIC2015-201, TIC201--202, TIC2015-203, TIC2015- TIC2015-204 and TIC2015-210 were prepared by extraction of approximately 2 cm^3^ pieces from frozen sponge material with 12 mL MeOH, *T. pedunculata* extracts were diluted approximately ten-fold with MeOH due to intense sample coloration. The extracts were filtered (0.2 μm) and analyzed at injection volumes of 2 and 5 μL, respectively. *T. nguni* samples were prepared by extraction of frozen sponge material with DCM/MeOH (2/1, *v*/*v*) followed by drying under reduced pressure. Samples were then resuspended in MeOH at 1 mg/mL, filtered (0.2 μm) and analyzed using injection volumes of 5 μL. All remaining samples were prepared by extracting frozen sponge material with DCM/MeOH (2/1, *v*/*v*) followed by drying under reduced pressure. Extract material was then redissolved in MeOH at 10 mg/mL, filtered (0.2 μm) and analyzed using injection volumes of 1 μL. LC-MS/MS data was recorded on a Bruker Compact QToF using an ESI-source in positive mode coupled to a Dionex Ultimate 3000 chromatograph using a gradient mobile-phase program employing water and acetonitrile as previously described [30]. The ESI-source parameters were set as follows in positive mode: end plate offset 500 V, capillary voltage 4500 V, nebulizer pressure 3.0 bar, dry gas flow 9.0 L/min, dry temperature 220 °C). An *m*/*z* window of 80 to 2000 was analyzed and MS^2^ spectra were recorded in data-dependent acquisition mode, selecting the three most intense precursor ions for acquisition of MS^2^ spectra at a quadrupole isolation width of 1.5 *m*/*z*, collision energy of 40 eV and transfer time of 80 μs.

### 4.3. Data Processing and Molecular Networking Analysis

After acquisition, the raw data were converted to mzXML format using the Bruker Compass software (Bruker, Bremen, Germany). The resulting mzXML files were processed in MZmine 2 (ver. 2.40) [36]. Mass lists were created using the mass detection module with a noise level of 1000 counts for MS^1^ and 40 counts for MS^2^. The chromatogram builder module was used to create peak lists with a minimum retention time of 0.05 min, a minimum peak height of 5000 counts and an *m*/*z* tolerance of 0.02. The peak lists were deconvoluted using the local minimum search algorithm with the following parameters: chromatographic threshold 0.01%, search minimum in retention time range 0.2 min, minimum relative height 0.1%, minimum absolute height 5000 counts, minimum ratio of peak top/edge 2, and peak duration range 0.02–4 min. For MS^2^ scan pairing, the *m*/*z* range was set to 0.02 and the retention time range to 0.5 min. Next, the deconvoluted peak lists were aligned using the Join aligner module with an *m*/*z* tolerance of 0.05, a retention time tolerance of 1.5 min and weighting for *m*/*z* and retention time both set to 100.

This ‘globally’ aligned peak list was then subjected to gap-filling and filtered to exclude all features present in the solvent control samples, as well as those without associated MS^2^ spectra and peak IDs were reset, followed by export of a .csv table and .mgf file.

In the next step, the *m*/*z* values for each feature in the .csv table were recalculated to represent the *m*/*z* value of the monoisotopic, singly-protonated precursor compound, by subtracting two neutron masses from the *m*/*z* value for each ^81^Br contained in a particular feature. Charge state and bromination/chlorination of ion features were determined through inspection of isotope distributions at the MS^1^-level. The *m*/*z* values of doubly-charged features were multiplied by two and one proton mass was subtracted from the result, so that singly- and doubly-charged states of the same precursor compound were now both assigned the *m*/*z* value of the singly-charged species. Finally, the .mgf file and the adjusted .csv file were uploaded to GNPS [37,38] and a job was submitted using the feature molecular networking workflow with the following parameters: Precursor and fragment ion mass tolerance = 0.02 Da, minimum cosine = 0.7, minimum matched fragment ions = 6, maximum number of edges per node = 6, minimum peak intensity = 40. The data was filtered by removing all product ions within +/− 17 Da of the precursor *m*/*z*. The files were normalized to compensate for differences in sample preparation and concentrations (row sum normalization) and the aggregation method for peak abundances per group was set to mean. The molecular network was visualized in Cytoscape (ver.3.7.1) [49] and the relative abundances of features were averaged for each of the sponge types.

## Data Availability

28S rRNA sequence data is available on Genbank and accession numbers are provided in Table S1. The completed molecular networking job can be accessed online at https://gnps.ucsd.edu/ProteoSAFe/status.jsp?task=a1dd30ef710c4fcdaf6872f023c134fb.

## Supplementary Materials

The following are available online at https://www.mdpi.com/1660-3397/19/2/68/s1, Table S1: Sponge metadata. Supplementary Materials S1: Isolation and characterization of makaluvamine C and 3-dihydrodiscorhabdin C. Figure S2.1: Molecular network of *T. nguni* and *T. favus*., Figure S2.2: Molecular network of *T. pedunculata*., Figure S2.3: Molecular network of *T. michaeli*, Figure S2.4: Molecular network of *C. bellae* and *L. apicalis*, Figure S2.5: Non-pyrroloiminoquinone clusters and singleton nodes belonging to the combined molecular network shown in Figure 2, Figure S3.1–S3.22: Additional MS^2^ spectra associated with the combined molecular network in Figure 2.
