# Supplementary material for: Unlocking the Diversity of Pyrroloiminoquinones Produced by Latrunculid Sponge Species
Source: Mar Drugs. 2021 Jan 28;19(2):68. doi: 10.3390/md19020068 (PMC7912287; doi:10.3390/md19020068)
Supplement: Supplementary file 1 [file marinedrugs-19-00068-s001.zip › Supplementary Materials/Supplementary Materials-S1.pdf]

Supplementary Materials

S1. Isolation of makaluvamine C from C. bellae

Frozen sponge material of a *C. bellae* specimen (TIC2016-053) was extracted with MeOH–DCM (1:2, v/v) and concentrated *i. vac.*. The sample was backloaded onto HP20, washed with water, eluted with MeOH and dried *i. vac.*. Extract material was resuspended in MeOH and fractionated into 12 fractions on Sephadex LH-20 (H_2O–MeOH–FA, 70:30:0.05, v/v/v). Fractions 3 to 6 were united, dried *i. vac*. and dissolved in MeOH, followed by RP-SPE (10 g C-18, H_2O–MeOH–FA, 80:20:0.05, v/v/v) to yield six fractions, with the first fraction containing makaluvamine C. The ^{1}H-NMR spectrum (A1.1) was consistent with literature reports [14] and the HMBC (A1.2) confirmed the position of the methyl group. Mass spectrometric analysis was performed in the same manner as described in section 4. *Materials and Methods*, indicated a molecular ion formula of $[C_{11}H_{12}N_3O]^+$ (m/z_{exp}=202.0930, m/z_{calc}=202.0980) and revealed a characteristic dominant methane neutral loss (A1.3). NMR spectra were obtained on a Bruker Avance DRX-600 MHz spectrometer (Bruker, Rheinstetten, Germany).

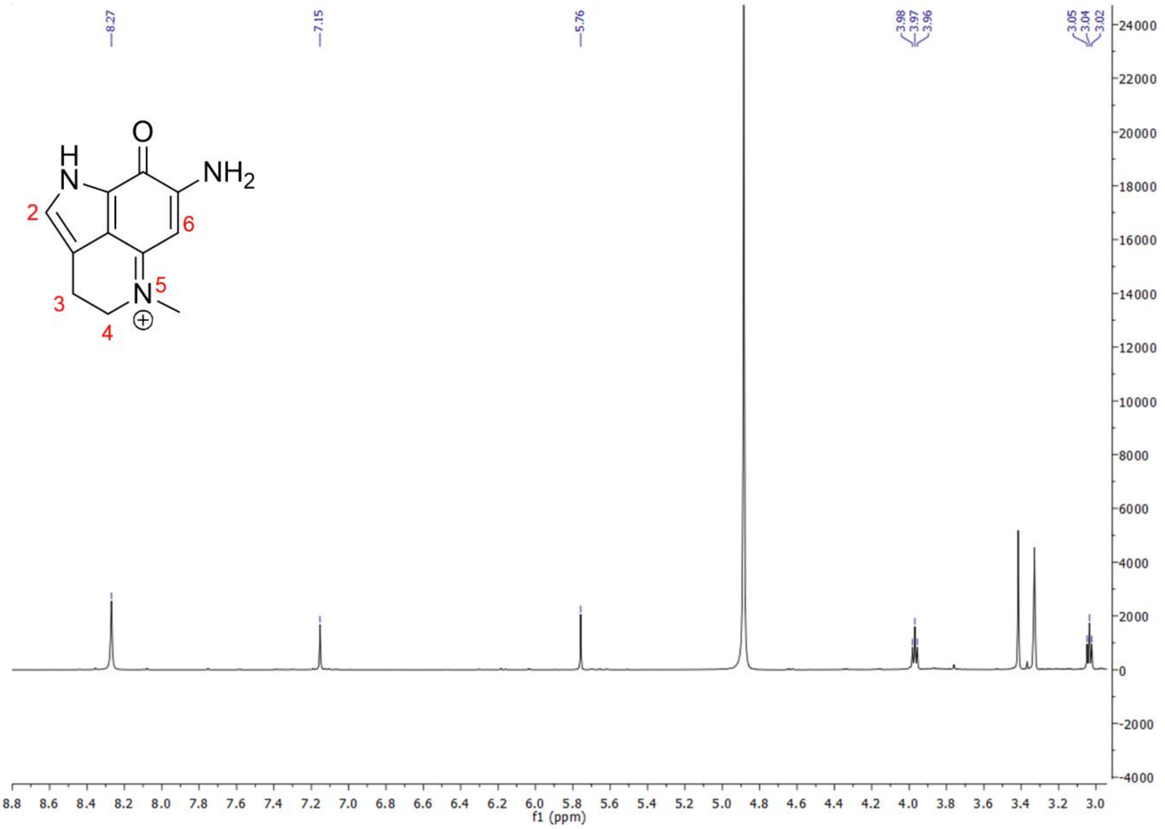

S1.1: ^{1}H-NMR spectrum of the makaluvamine C isolate in CD_3OD.

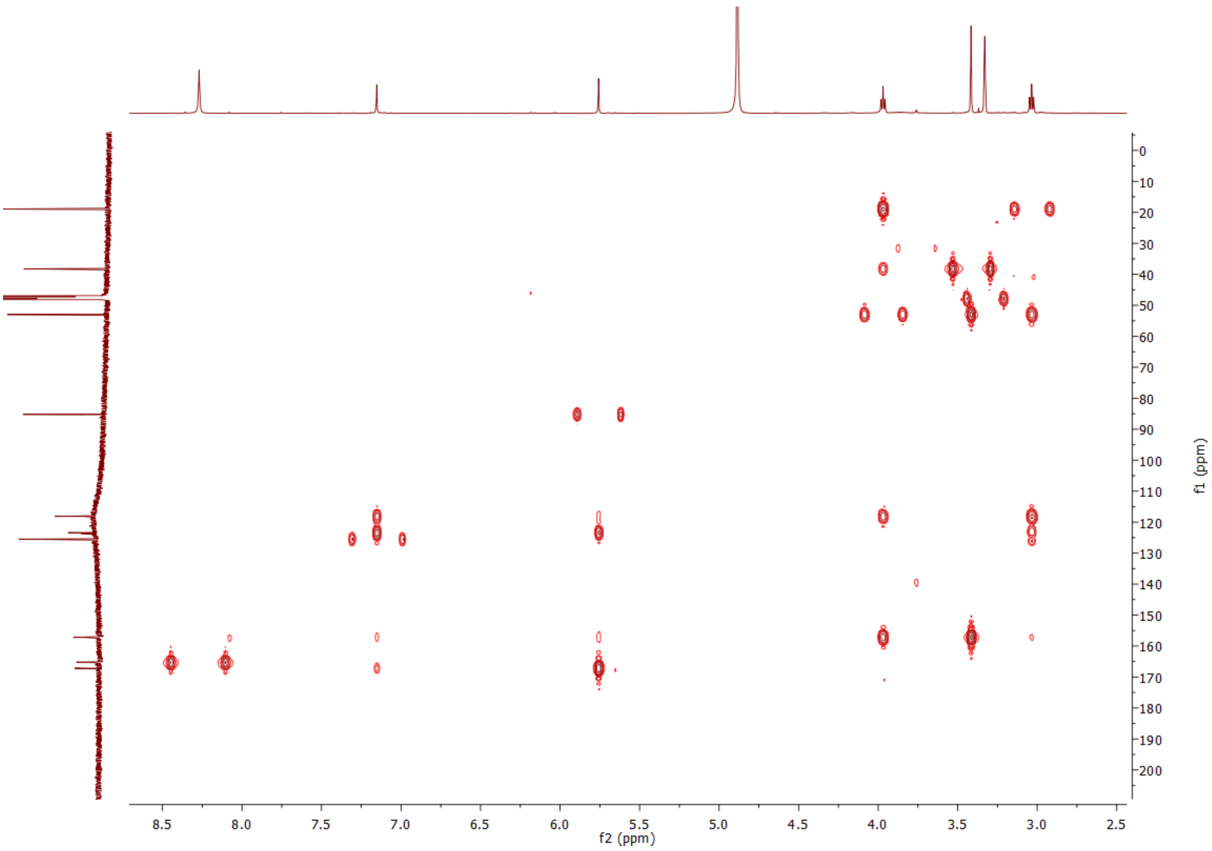

S1.2: HMBC spectrum of the makaluvamine C isolate in CD_3OD.

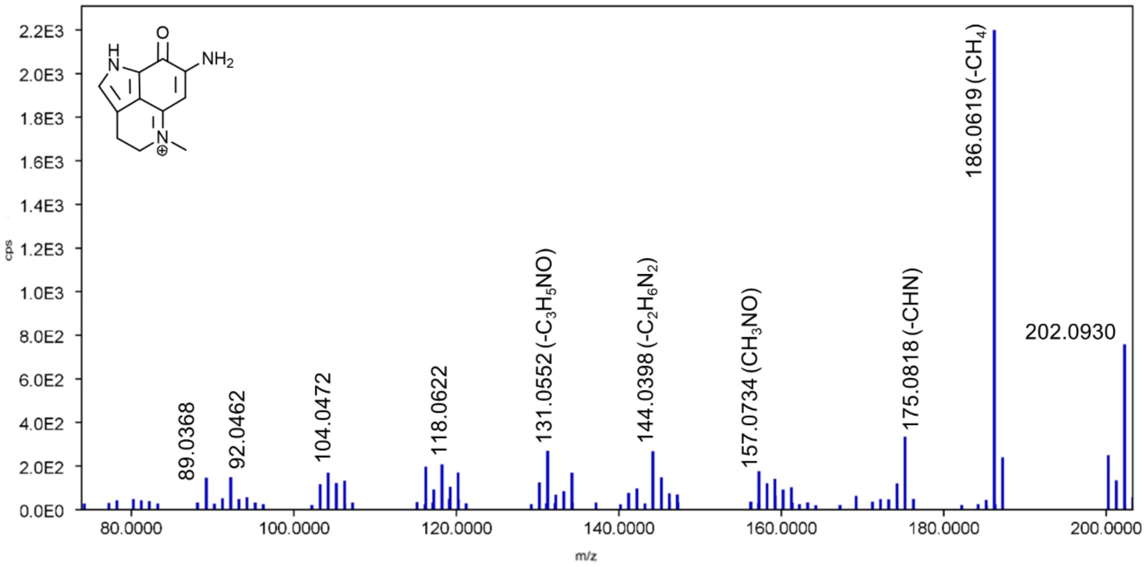

S1.3: MS^2 spectrum (collision energy = 40 eV; LC-MS/MS mode) of the makaluvamine C isolate.

Isolation of 3-dihydrodiscorhabdin C from *T. pedunculata*

Frozen sponge material of a *T. pedunculata* specimen (TIC2015-216) was extracted with MeOH–DCM (1:2, v/v) and dried *i.vac.*. Extract material was resuspended in MeOH and separated through RP-SPE (10 g C-18, H_2O-MeOH-FA, 60:40:0.05, v/v/v). One major fraction was selected as a dark red band and dried *i. vac.*. This fraction was dissolved in MeOH and again subjected to RP-SPE (2 g C-18, H_2O-MeOH-FA, 70:30:0.05, v/v/v) to yield 3-dihydrodiscorhabdin C as a dark red solid. ^1H-NMR, COSY and

HMBC data (A2.1-A2.3) were consistent with published values [1] and were acquired using a Bruker Avance DRX-600 MHz spectrometer (Bruker, Rheinstetten, Germany).

Mass spectrometric analysis was carried out in direct injection mode of a MeOH solution using the same ESI-QToF parameters as described in section 4. *Materials and Methods.* The MS^2 spectrum (A2.4) confirmed the expected *m/z* value of the $[M+H]^+$ ion corresponding to an ion formula of $[C_{18}H_{16}Br_2N_3O_2]^+$ (m/z_{exp}=463.9600, m/z_{calc}=463.9609) and showed a prominent neutral loss of H_3BrO (*m/z* 366) and all other significant neutral losses were found to incorporate bromine and oxygen. A putative fragmentation mechanism is shown in A2.4 beginning with proton migration, inductive cleavage of water and a ring expansion (RE). This is proposedly followed by elimination of hydrogen bromide and combined hydrogen migration and rearrangement driven by aromatization to produce a highly conjugated product ion at *m/z* 366.0272. The second most (but much less) intense product ion at *m/z* 287.1082 corresponds to neutral loss of an additional bromine radical, however, the preferred ion structure and whether it is in fact formed along the same pathway as the *m/z* 366.0272 fragment are still unknown and the fragmentation mechanism of this compound is subject of ongoing investigation.

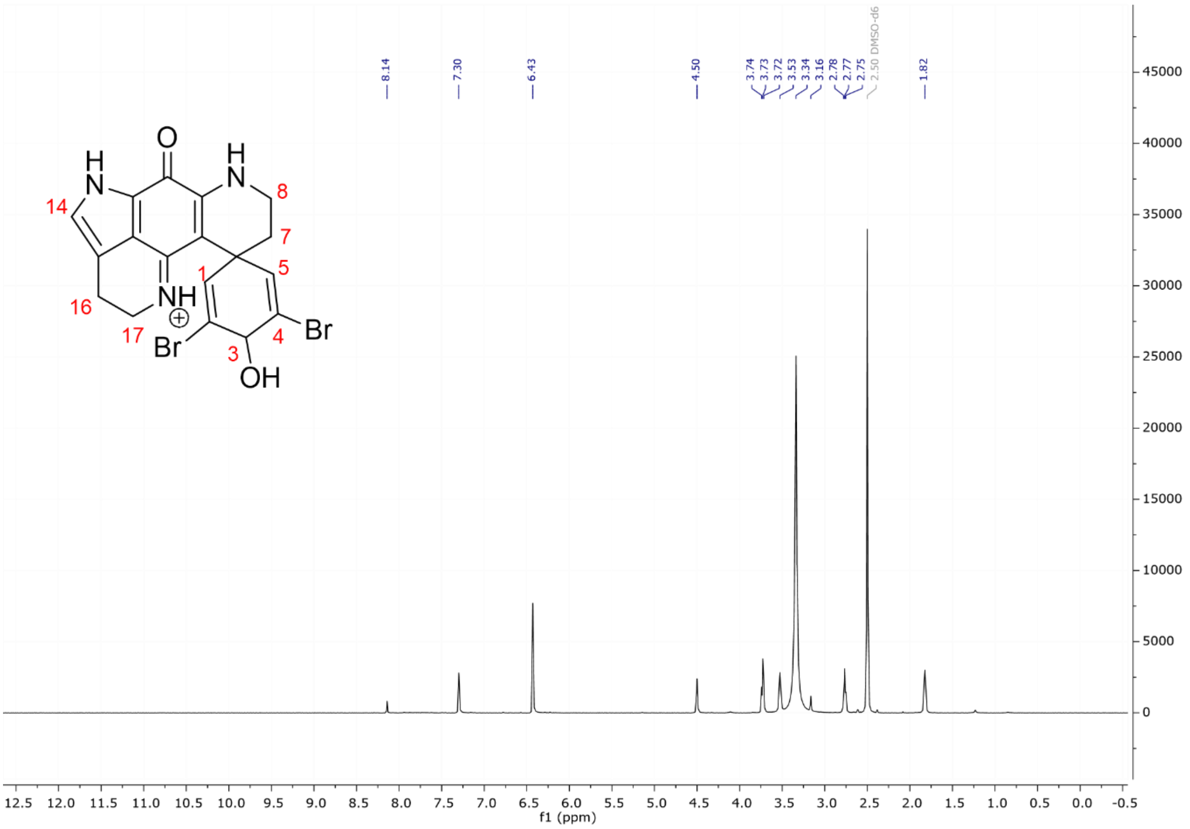

S1.4: ^{1}H-NMR spectrum of 3-dihydrodiscorhabdin C in DMSO-d_6.

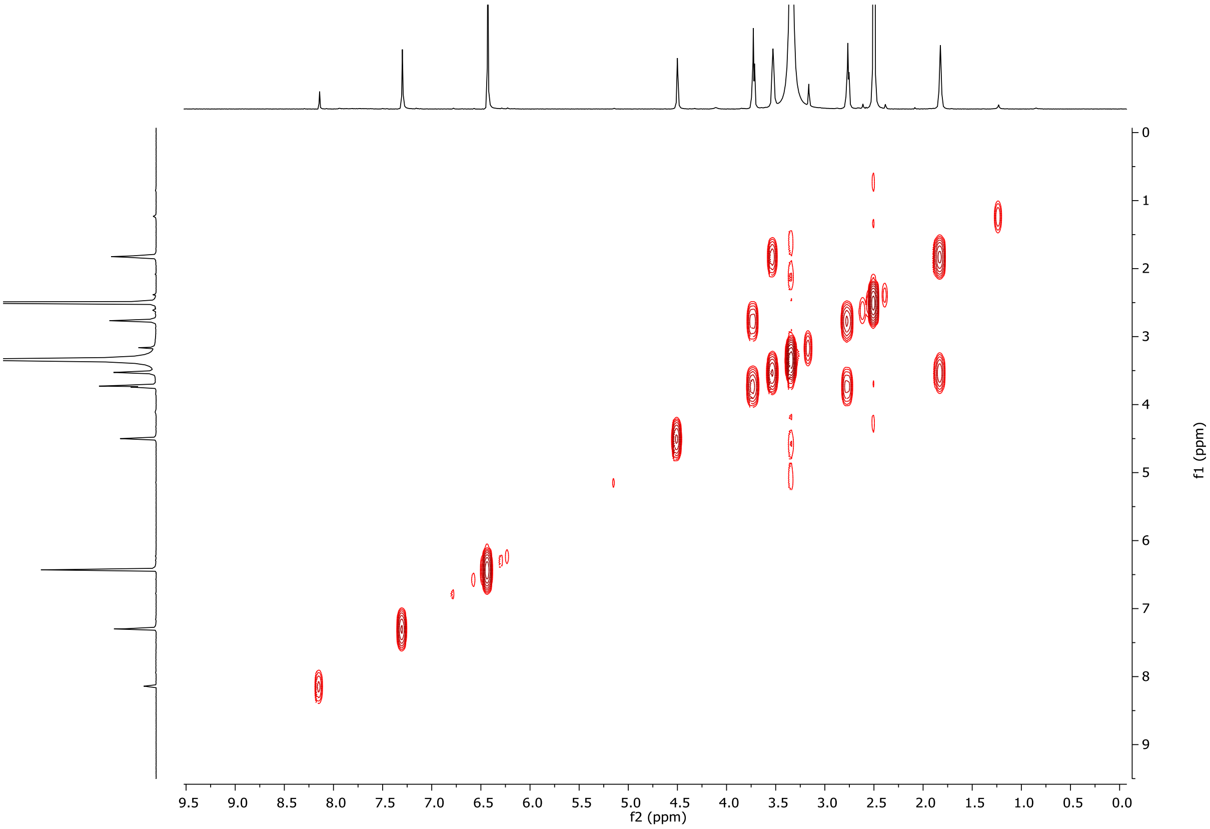

S1.5: COSY spectrum of 3-dihydrodiscorhabdin C in DMSO-d6.

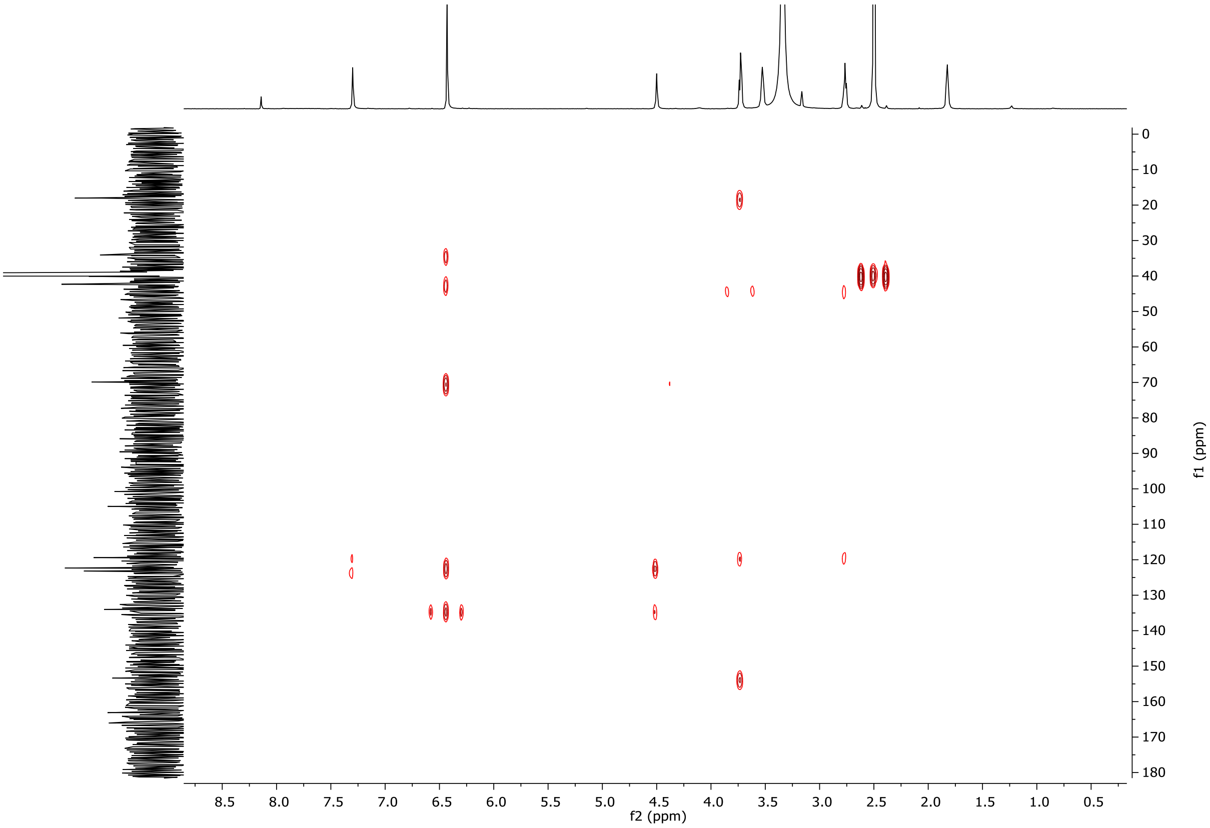

S1.6: HMBC spectrum of 3-dihydrodiscorhabdin C in DMSO-d6.

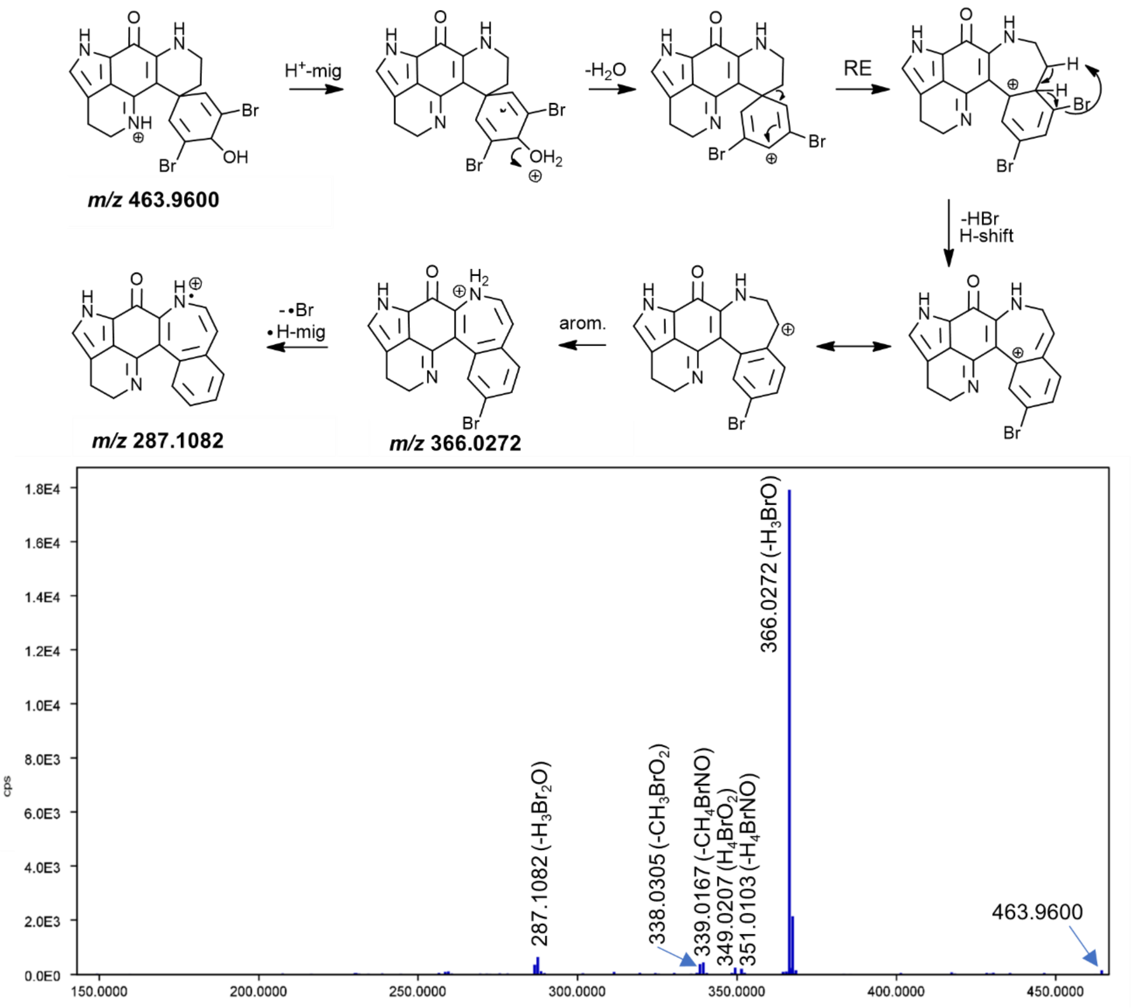

S1.7: MS^2 spectrum (collision energy = 40 eV; direct injection mode) of the monoisotopic precursor ion of 3-dihydrodiscorhabdin C (463.96 Da) and proposed fragmentation reactions.

Reference

1. Copp, B.R.; Fulton, K.F.; Perry, N.B.; Blunt, J.W.; Munri, M.H.G. Natural and Synthetic Derivatives of Discorhabdin C, a Cytotoxic Pigment from the New Zealand Sponge *Latrunculia* cf. bocagei. *J. Org. Chem.* **1994**, *59*, 8233–8238, doi:10.1021/jo00105a047.
