# Supplementary material for: Unlocking the Diversity of Pyrroloiminoquinones Produced by Latrunculid Sponge Species
Source: Mar Drugs. 2021 Jan 28;19(2):68. doi: 10.3390/md19020068 (PMC7912287; doi:10.3390/md19020068)
Supplement: Supplementary file 1 [file marinedrugs-19-00068-s001.zip › Supplementary Materials/Supplementary Materials-S2.pdf]

S2: Molecular networks of data subsets (S2.1-S2.4) and non-pyrroloiminoquinone clusters and singleton nodes belonging to the molecular network shown in Fig. 2 (S2.5).

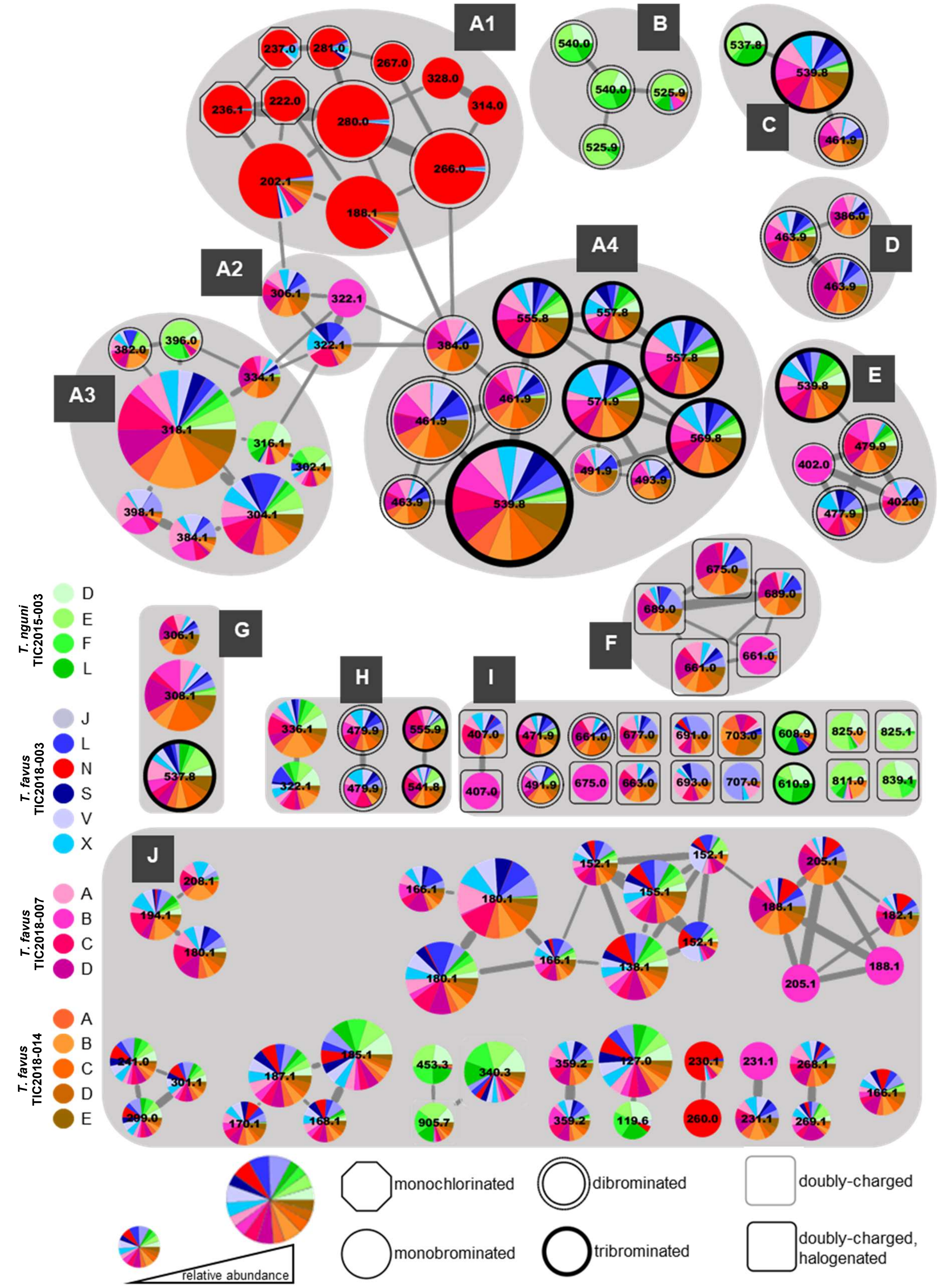

S2.1: Molecular Network of *T. nguni* and *T. favus* (min. cosine = 0.7). **A1:** Unbranched makaluvamines and damirones; **A2:** Discorhabdin I and a hydroxylated derivative; **A3:** Tsitsikammamines; **A4:**

Brominated C-series discorhabdins; **B:** Oxygenated discorhabdin derivatives; **C:** 3-Dihydro-7,8-dehydrodiscorhabdins; **D:** 3-Dihydrodiscorhabdins; **E:** V-series discorhabdins; **F:** Ovothiol-substituted C-series discorhabdins; **G:** Branched makaluvamines and 14-bromo-16,17-dehydrodiscorhabdin C; **H:** Miscellaneous clusters of pyrroloiminoquinones and related compounds; **I:** Miscellaneous brominated singletons; **J:** Non-pyrroloiminoquinones. (https://gnps.ucsd.edu/ProteoSAFe/status.jsp?task=3479207be99841bd8ed4092c5cbb0bab)

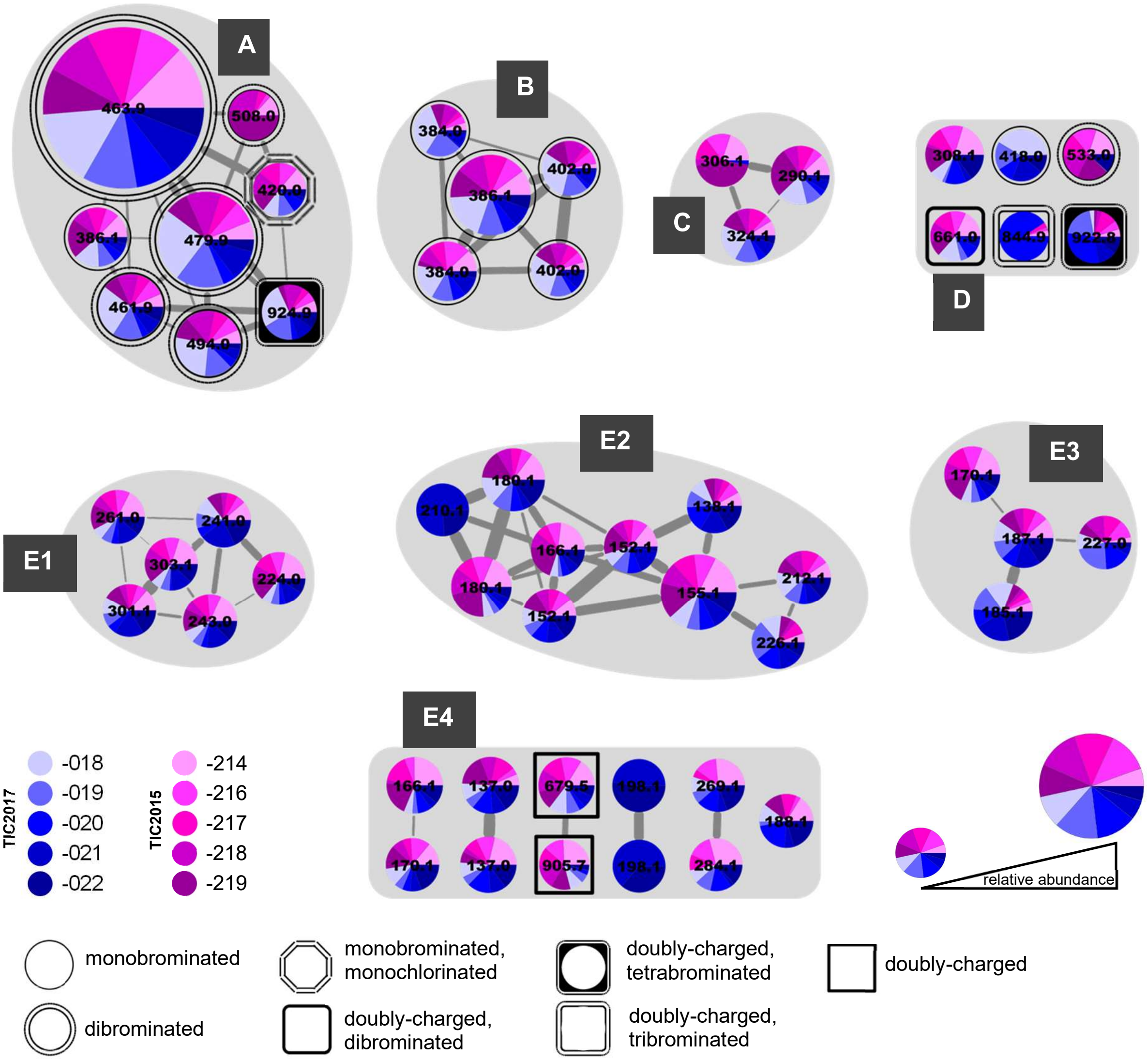

S2.2: Molecular Network of *T. pedunculata* (min. cosine = 0.6). **A:** 3-Dihydrodiscorhabdins of the C-series; **B:** C-3 carbonyldiscorhabdins of the C- and V-series; **C:** Discorhabdin I and Derivatives; **D:** Miscellaneous pyrroloiminoquinone singletons; **E1-E4:** Non-pyrroloiminoquinone Compounds. (https://gnps.ucsd.edu/ProteoSAFe/status.jsp?task=30bb99d64d594558a324bb342cf1008d)

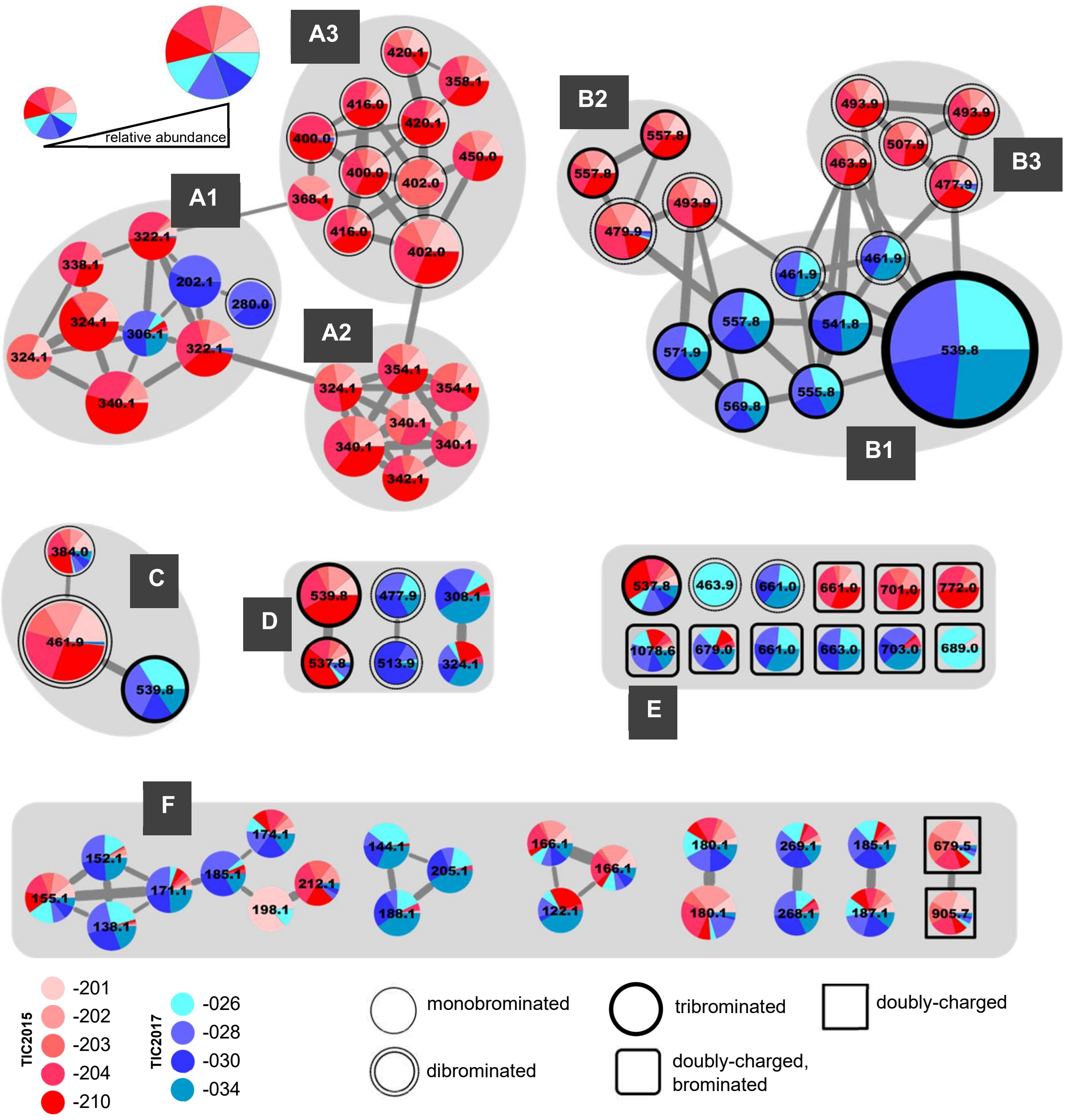

S2.3: Molecular Network of extracts from *T. michaeli* (min. cosine = 0.7). **A1:** Makaluvamines and discorhabdins; **A2:** New hydroxylated discorhabdins; **A3:** New C-series discorhabdins; **B1-B3:** Multibrominated C-series discorhabdins; **C:** 3-Dihydro-7,8-dehydrodiscorhabdins; **D:** Miscellaneous pyrroloiminoquinone clusters; **E:** Miscellaneous pyrroloiminoquinone singletons; **F:** Non-pyrroloiminoquinones.
(https://gnps.ucsd.edu/ProteoSAFe/status.jsp?task=5bd2a003af0a48f98a8e001ea837b222)

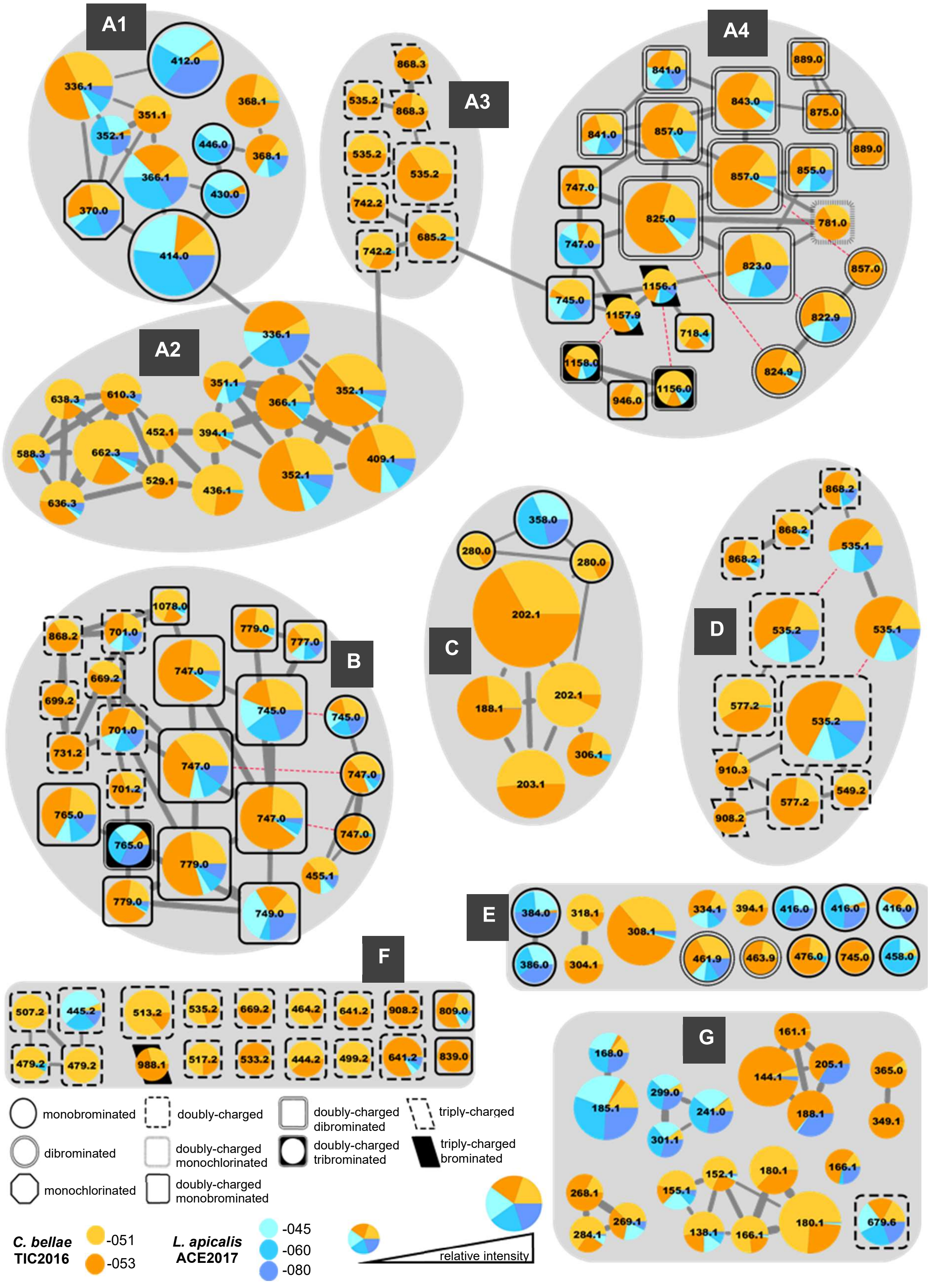

S2.4: Molecular Network of *C. bellae* and *L. apicalis* (min. cosine = 0.7). **A1:** Discorhabdins of the A- and D-series; **A2:** Discorhabdins of the D- and A-series; **A3:** Non-brominated pyrroloiminoquinone di- and trimers; **A4:** Thioether-bridged discorhabdin dimers; **B:** Disulphide-bridged discorhabdin di- and

trimers; **C:** Unbranched makaluvamines and related compounds; **D:** Ovothiol-substituted A/D-series discorhabdins; **E:** Other pyrroloiminoquinones; **F:** Multiply-charged clusters and singletons; **G**: Non-pyrroloiminoquinones. (https://gnps.ucsd.edu/ProteoSAFe/status.jsp?task=6ba31b7a4a114164ac71a7ecc1076435)

Note: The molecular networks shown in S2.1.-S2.4. were created from the same raw data and in the same manner as the molecular network in the main article (Fig. 2.) except for S2.2 for which a less stringent minimum cosine value (0.6) was selected due to the limited number of features.

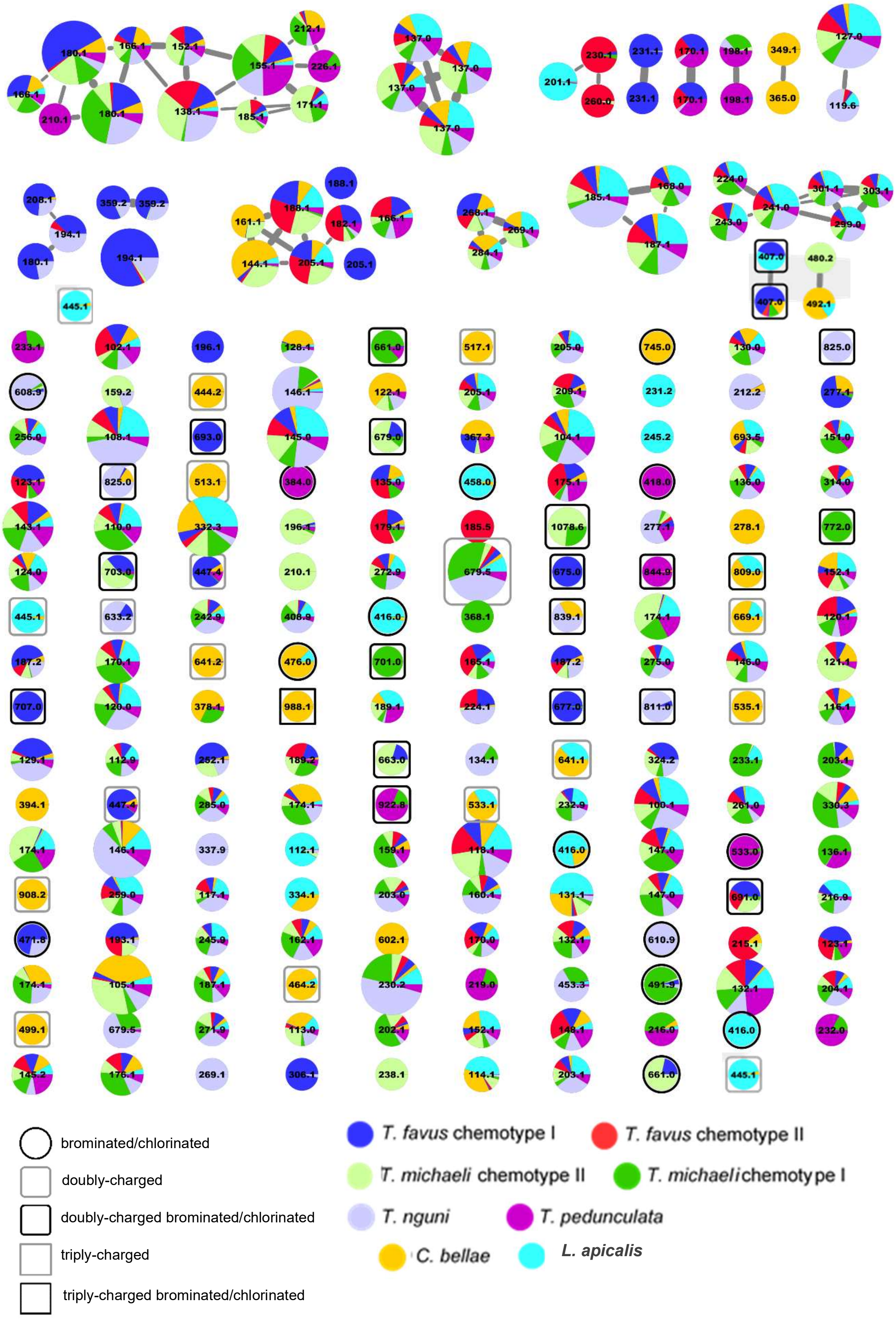

S2.5: Non-pyrroloiminoquinone clusters and singleton nodes belonging to the molecular network shown in Fig. 2.
