# Supplementary material for: Unlocking the Diversity of Pyrroloiminoquinones Produced by Latrunculid Sponge Species
Source: Mar Drugs. 2021 Jan 28;19(2):68. doi: 10.3390/md19020068 (PMC7912287; doi:10.3390/md19020068)
Supplement: Supplementary file 1 [file marinedrugs-19-00068-s001.zip › Supplementary Materials/Supplementary_Material_S3.pdf]

S3: Additional MS² spectra associated with the combined molecular network in Fig. 2.

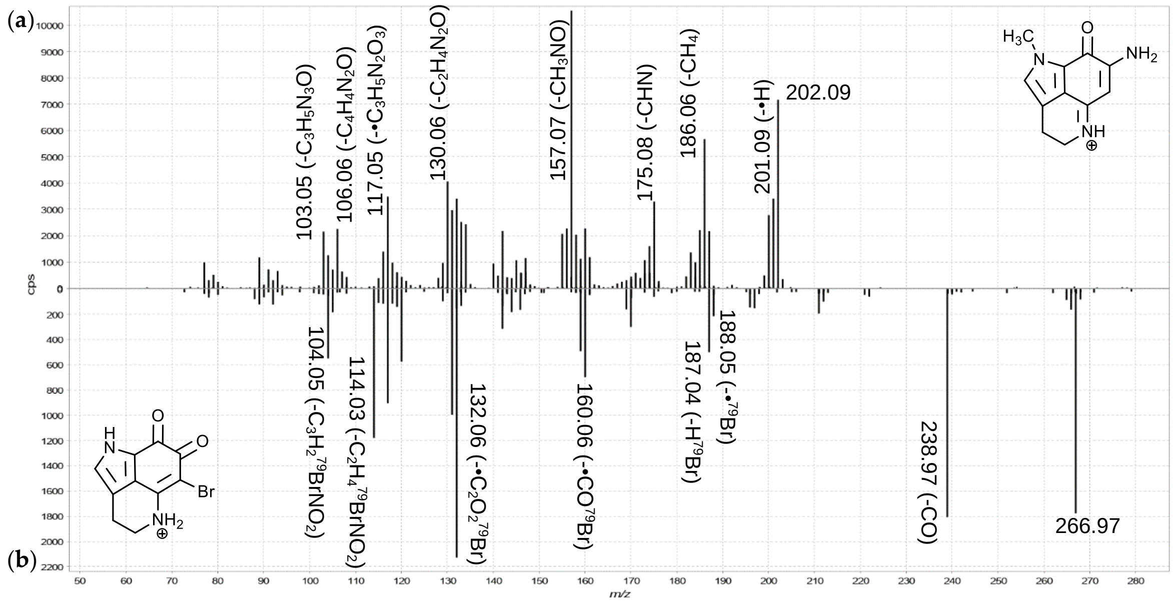

S3.1: MS^2 spectra of the 202.1 Da node (**a**, annotated as makaluvamine A) and the 267.0 Da node (**b**, annotated as makaluvamine O) from group A.

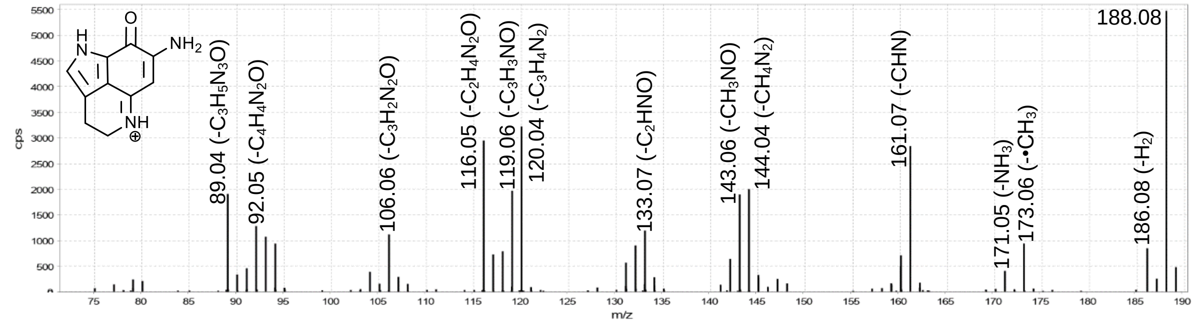

S3.2: MS^2 spectrum of the 188.0 Da node from group A (makaluvamine I).

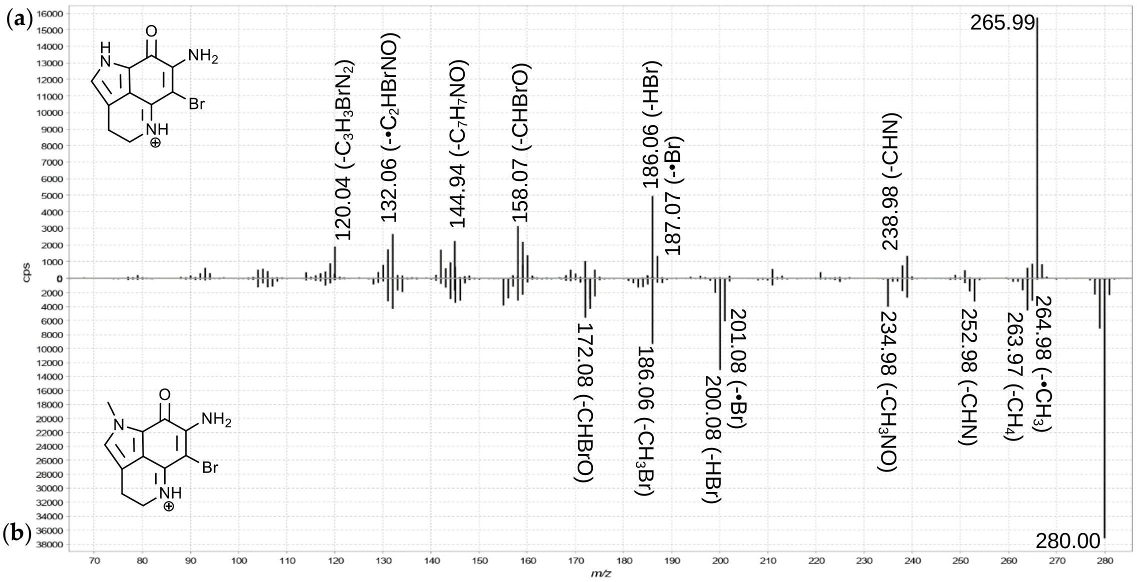

S3.3: MS^2 spectra of the 266.0 Da node (**a**, annotated as makaluvamine N) and the large 280.0 Da node (**b**, annotated as makaluvamine N) from group A.

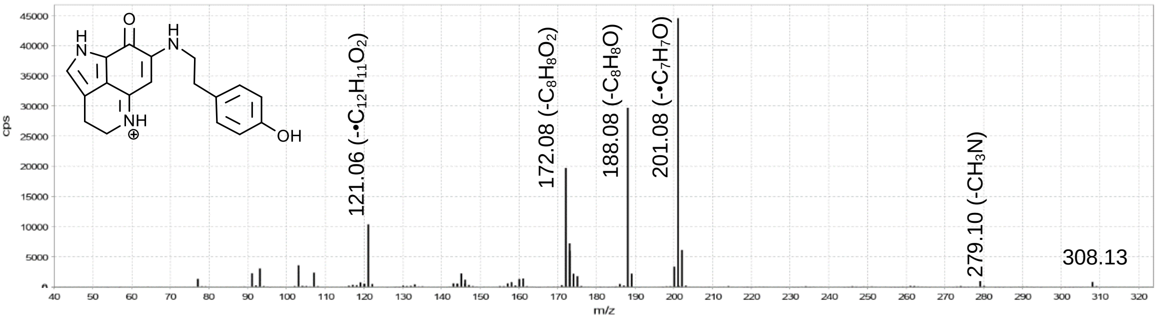

S3.4: MS^2 spectrum of the 308.1 Da node (annotated as makaluvamine D) from group A.

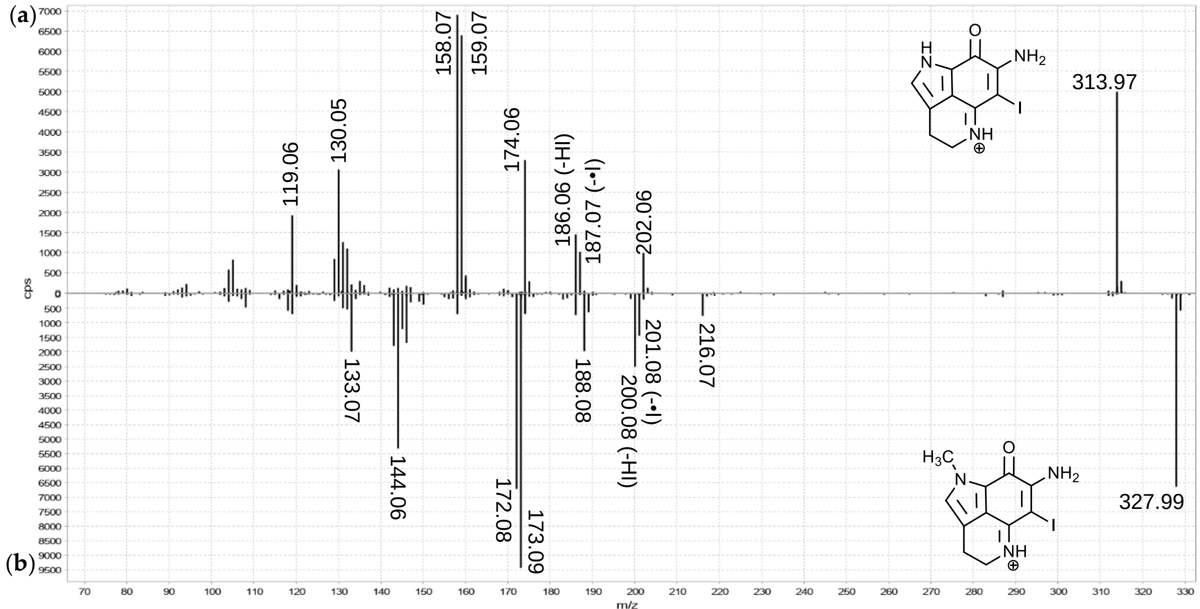

S3.5: MS^2 spectra of the 314.0 (**a**) and 328.0 Da (**b**) nodes from group A (putative iodinated makaluvamines).

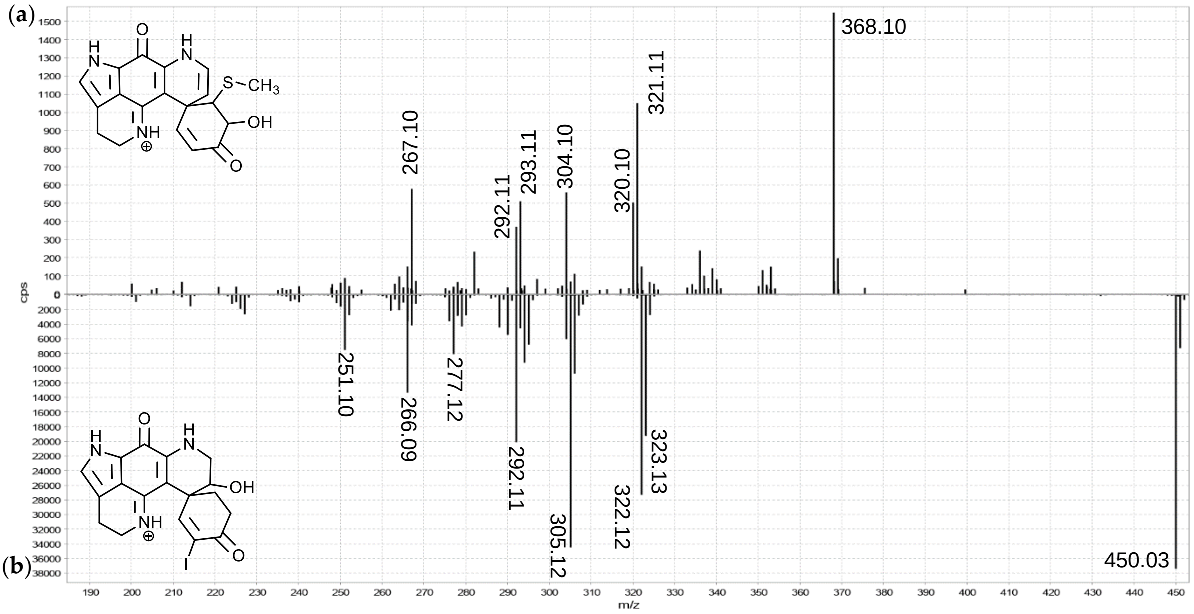

S3.6: MS[2] spectra of the 368.1 Da node (**a**, putative thiomethylated discorhabdin) and the 450.0 Da node (**b**, putative iodinated discorhabdin) from group C.

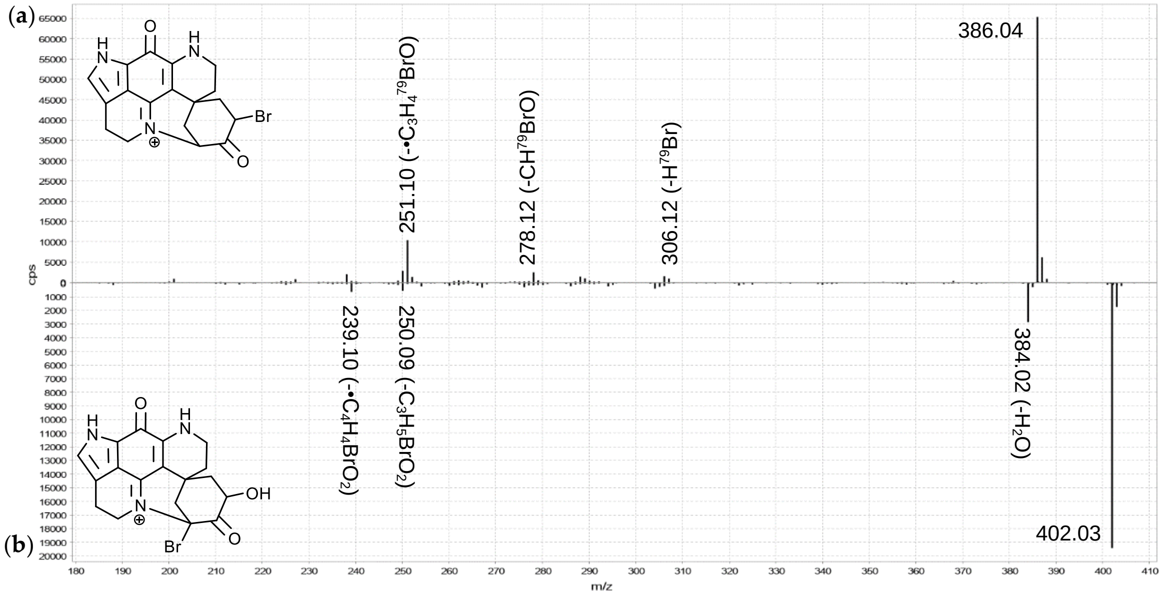

S3.7: MS[2] spectra of the purple 386.1 Da (**a**) and the rightmost 402.0 Da (**b**) node from group C (putative V-series discorhabdins).

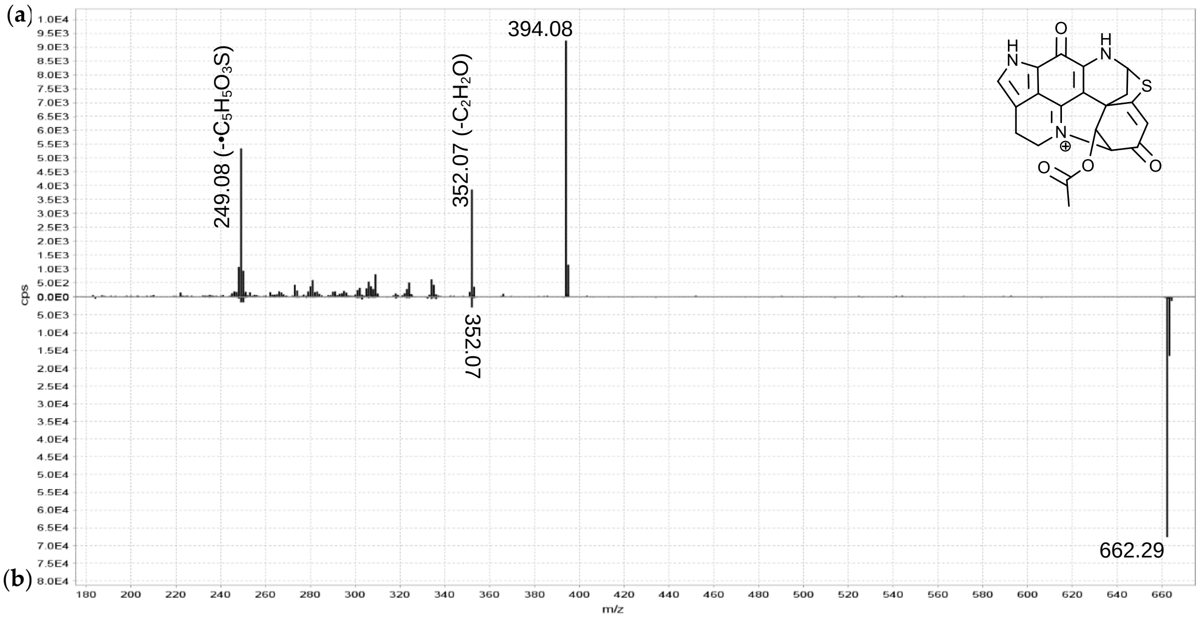

S3.8: MS[2] spectra of the 394.1 Da (**a**) and 662.3 Da (**b**) nodes from group D and annotated as discorhabdin L derivatives.

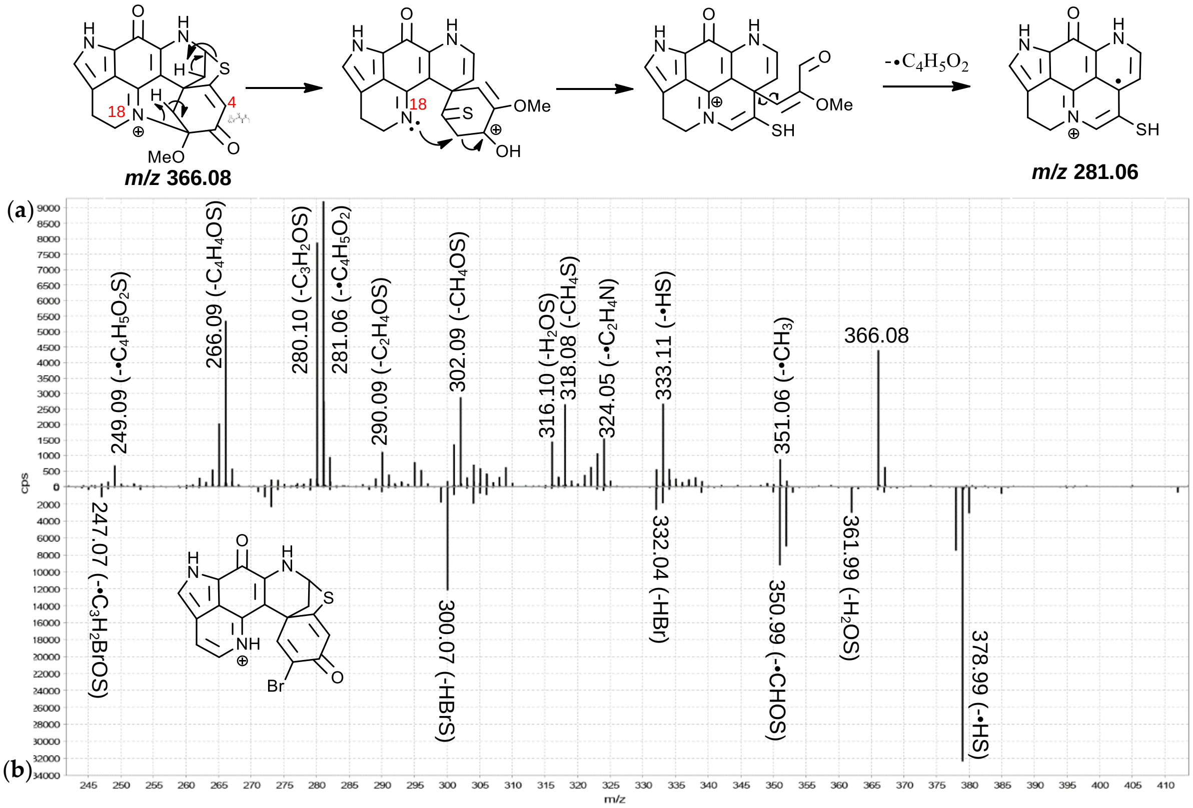

S3.9: MS[2] spectra of the 366.1 Da (**a**, annotated as a new 2-methoxydiscorhabdin D) and 412.0 Da (**b**, annotated as discorhabdin Q) nodes from group E.

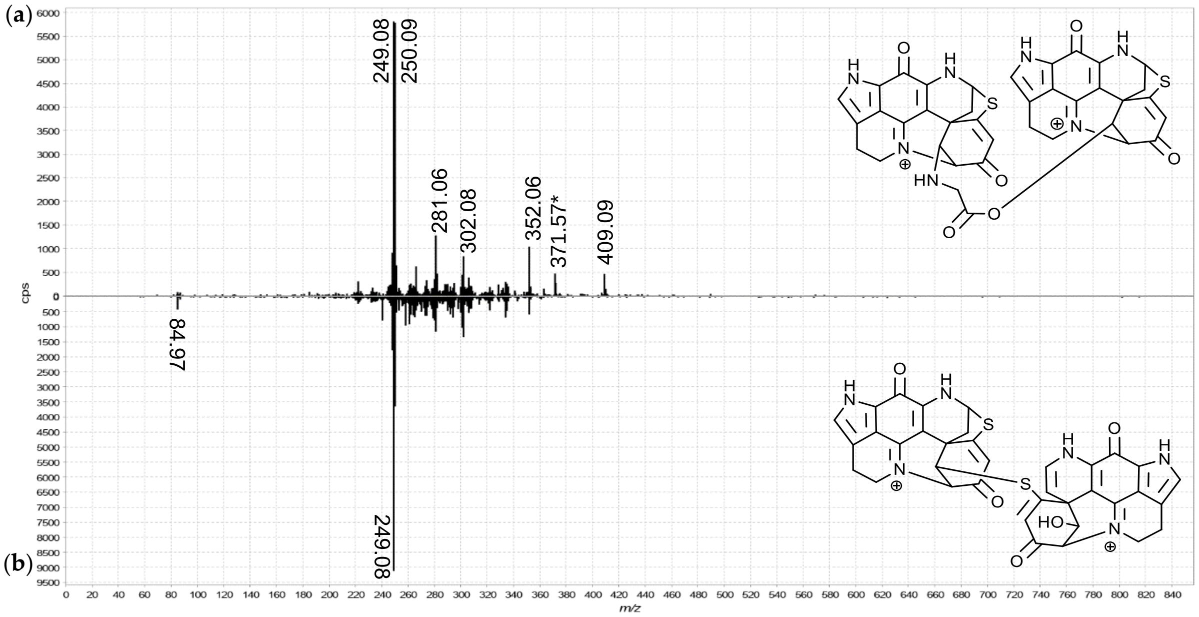

S3.10: Extended MS[2] spectra of the 742.2 Da (**a**, annotated as a glycine-linked discorhabdin dimer) and the 685.2 Da node (**b**, annotated as a discorhabdin dimer) from group F (Fig.7) (*= doubly-charged residual precursor ion).

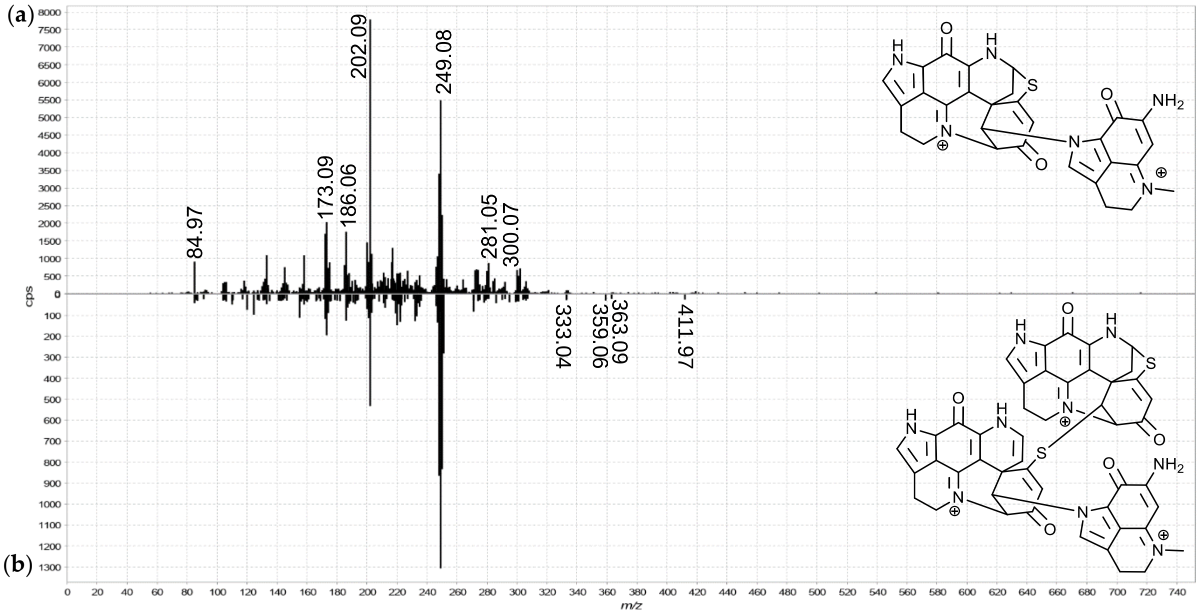

S3.11: Extended MS[2] spectra of the larger 535.2 Da (**a**, annotated as a discorhabdin-makaluvamine dimer) and the upper 868.2 Da node (**b**, annotated as a discorhabdin-discorhabdin-makaluvamine trimer) from group F (Fig.8).

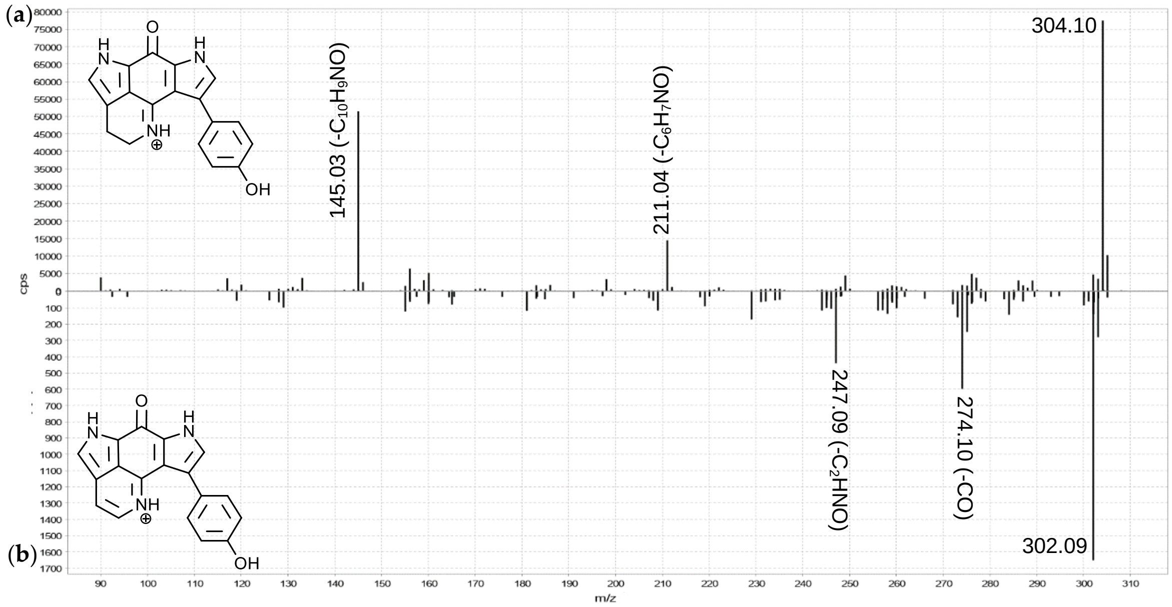

S3.12: MS^2 spectra of the 304.1 Da node (**a**, annotated as tsitsikammamine A) and the 302.1 Da node (**b**, annotated as 16,17-dehydrotsitsikammamine A) from group G.

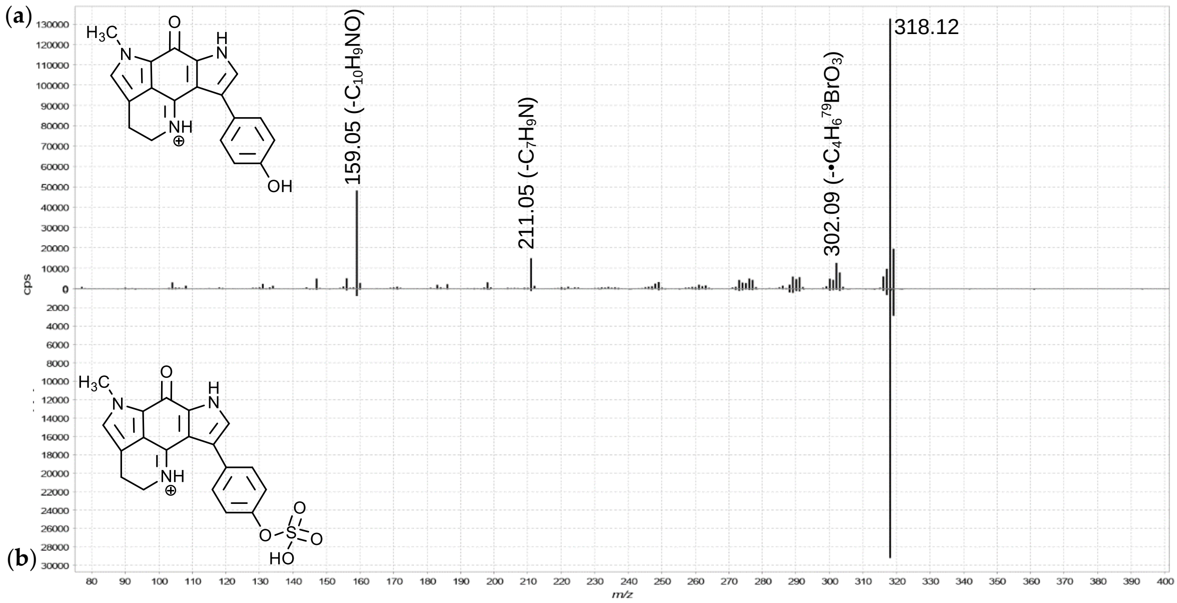

S3.13: MS^2 spectra of the 318.1 Da node (**a**, annotated as tsitsikammamine B) and the 398.1 node (**b**, putative sulfoxide derivative of tsitsikammamine B) from group G.

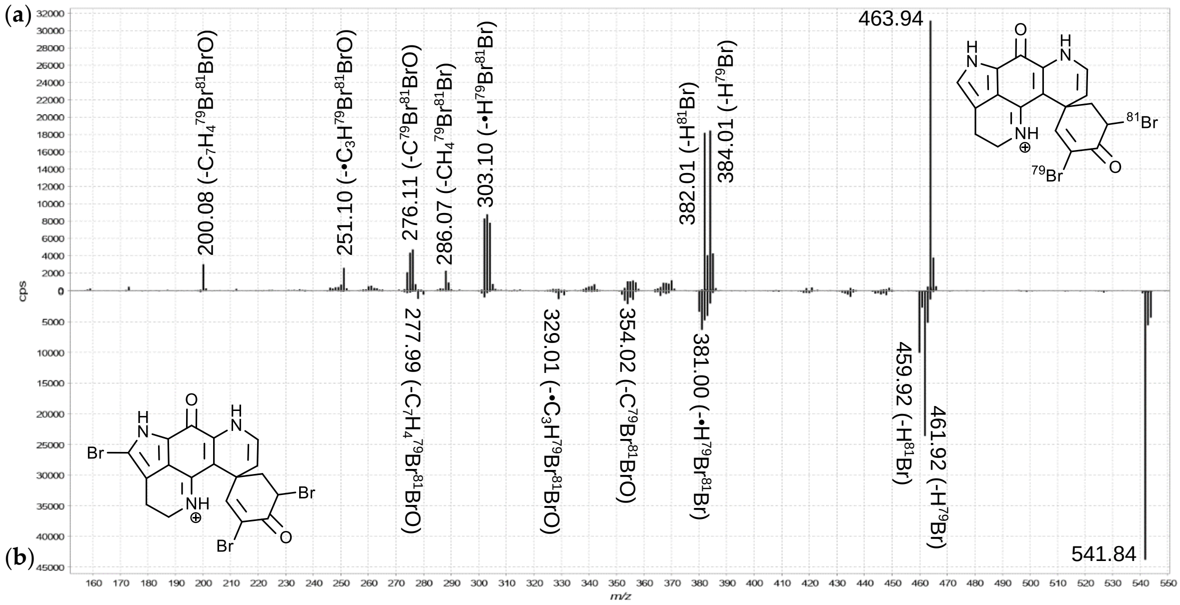

S3.14: MS^2 spectra of the leftmost 461.9 Da node (**a**, $^{79}Br^{81}Br$ precursor, annotated as a discorhabdin C regiomer) and the larger 539.8 Da node (**b**, $^{79}Br_2{}^{81}Br$ precursor, annotated as a 14-bromodiscorhabdin C regiomer) from group H.

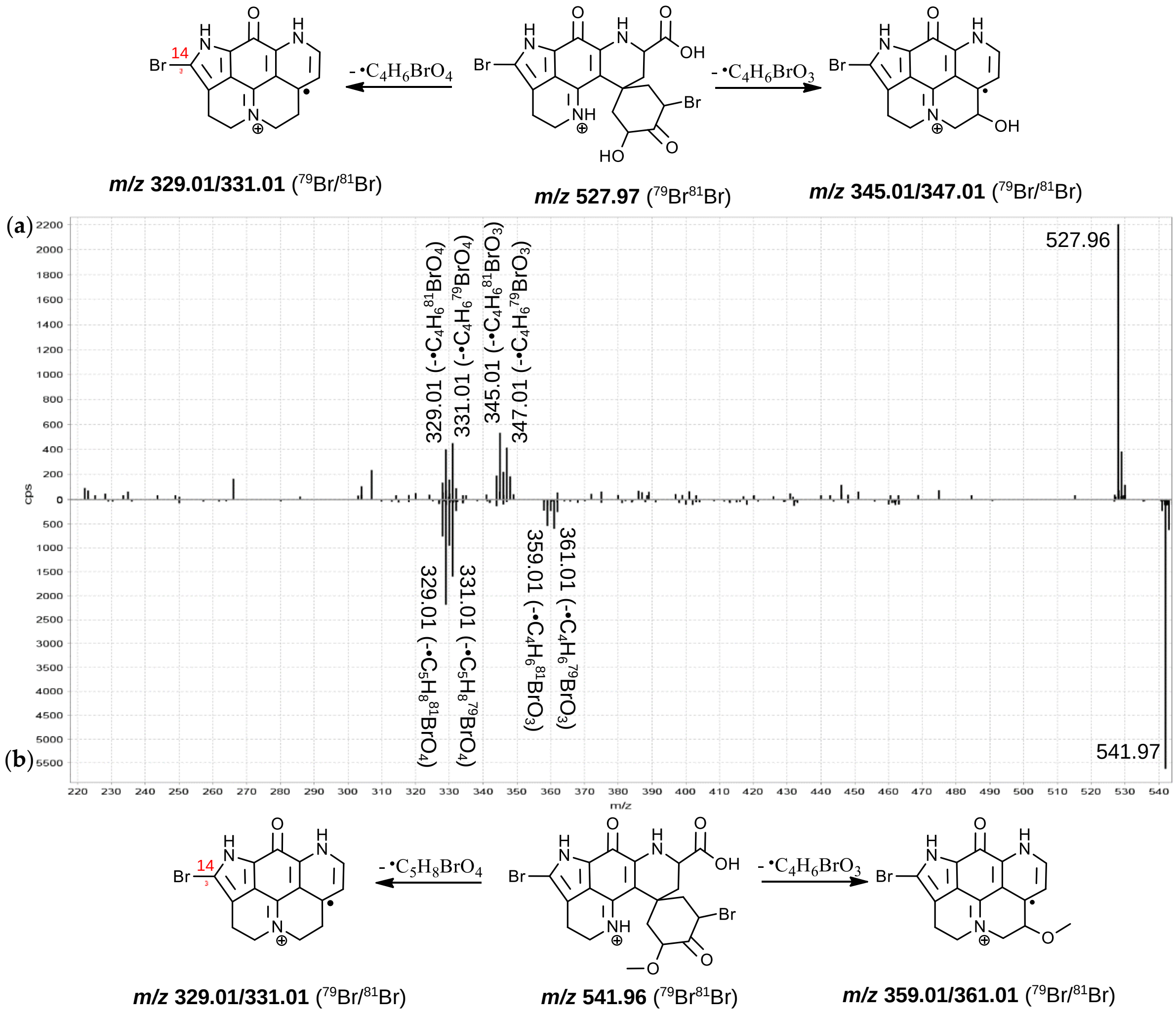

S3.15: MS^2 spectra ($^{79}Br^{81}Br$ isotopes) of the nodes at 525.9 (**a**) and 540.0 Da (**b**) from group I, putatively annotated as new discorhabdins and proposed fragment structures.

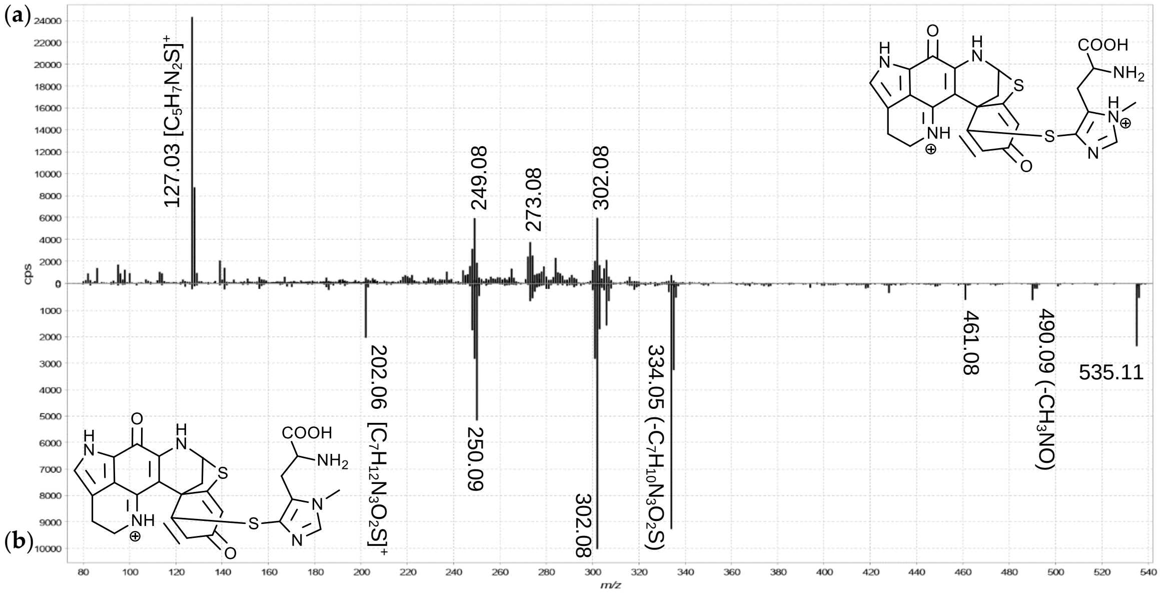

S3.16: MS[2] spectra of the smaller 535.1 Da pair of nodes (**a** doubly-charged, **b** singly-charged, annotated as discorhabdin G*) from group L.

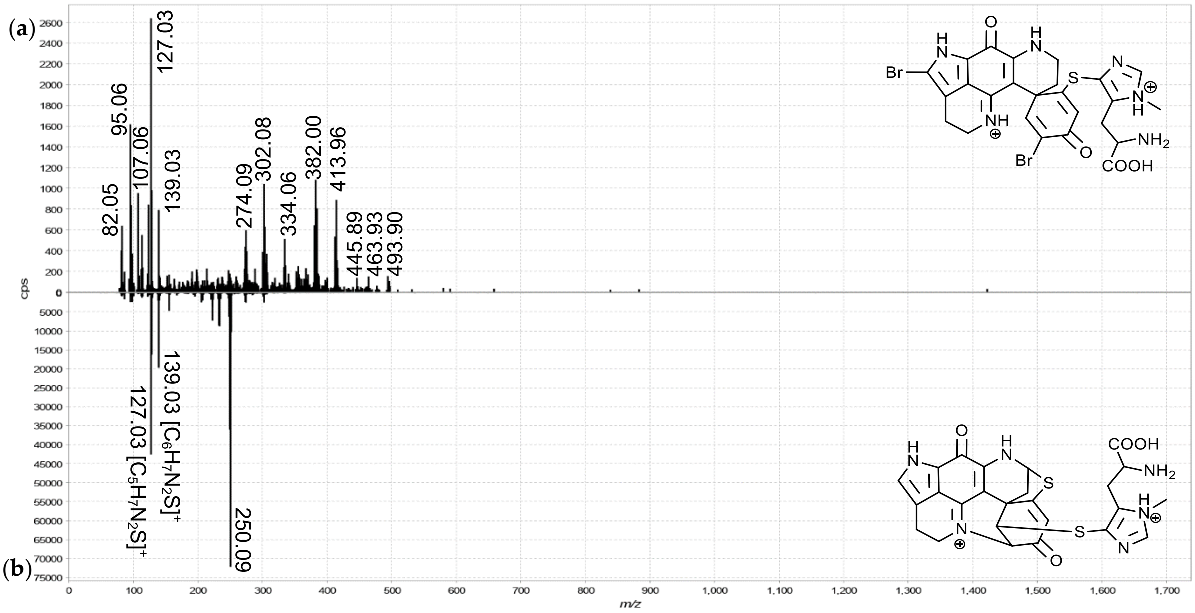

S3.17: Extended MS[2] spectra of the 661.9 Da node from group K (**a** doubly-charged, $^{79}Br^{81}Br$ precursor) and the larger 535.1 Da node from group L (**b** doubly-charged, annotated as discorhabdin H) from group L (Fig.11).

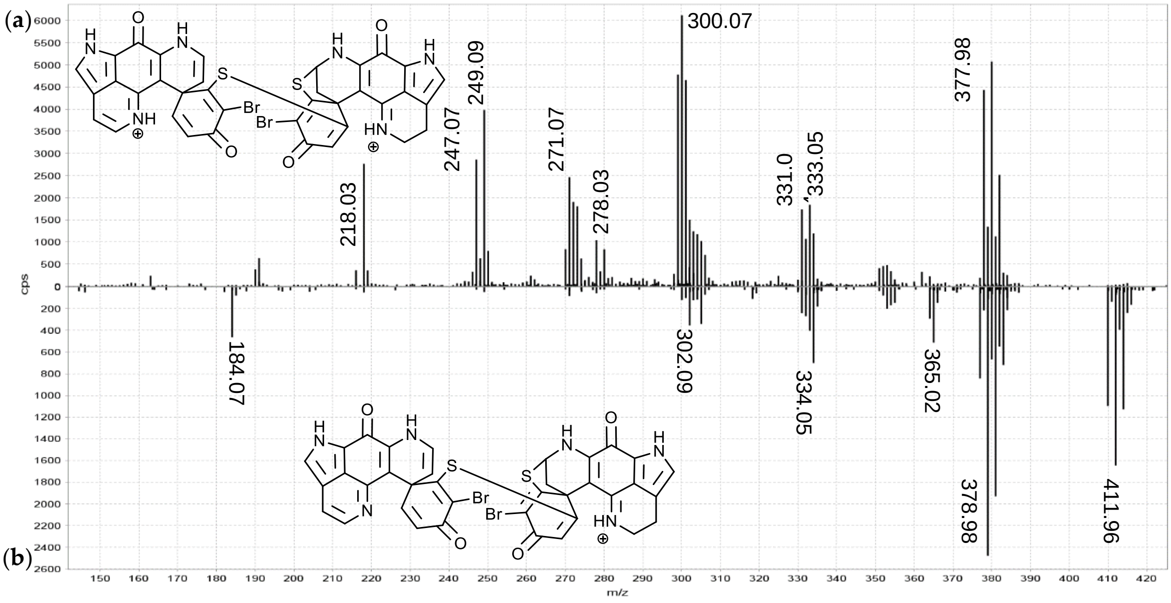

S3.18: MS[2] spectra of the doubly-charged 822.9 Da node (**a**) and the singly-charged 822.9 Da node (**b**) from group N, annotated as different charge-states of the same compound, a discorhabdin Q-discorhabdin B dimer.

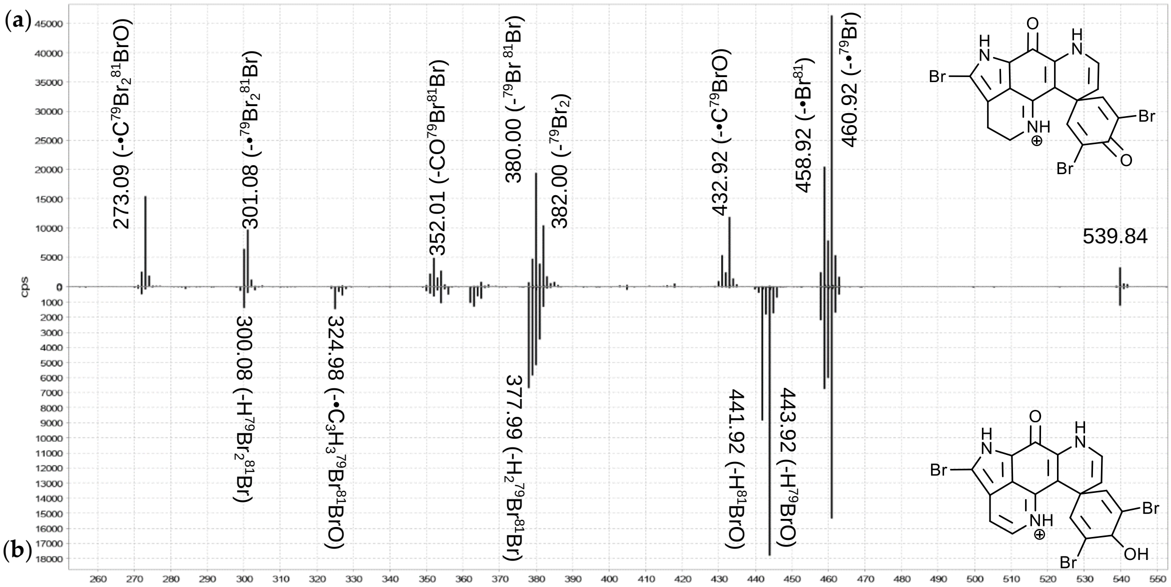

S3.19: MS[2] spectra of the large 537.8 Da singleton (**a** $^{79}Br_2{}^{81}Br$ precursor, annotated as 14-bromo-7,8-dehydrodiscorhabdin C) and the 537.8 Da node of the neighbouring cluster (**b**, $^{79}Br_2{}^{81}Br$ precursor) from group O.

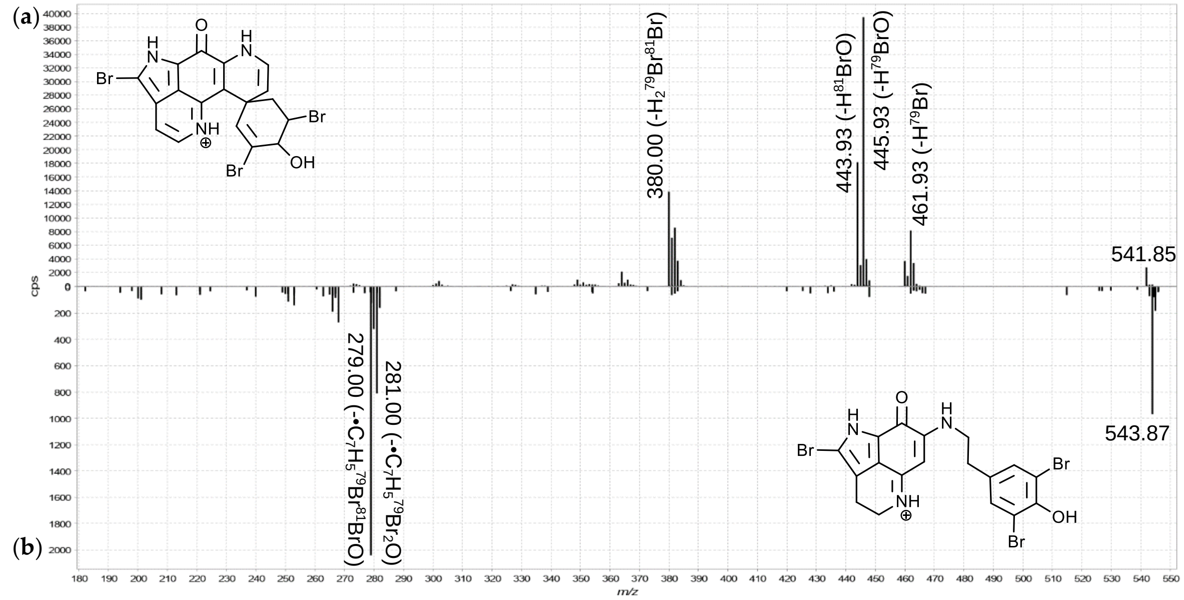

S3.20: MS[2] spectra of the 539.8 Da node (**a**) from the second cluster in group O annotated as a 14-bromo-3-dihydrodiscorhabdin C regioisomer and the 541.8 Da node (**b**) of the third cluster annotated as a new tribrominated analogues of makaluvamine D.

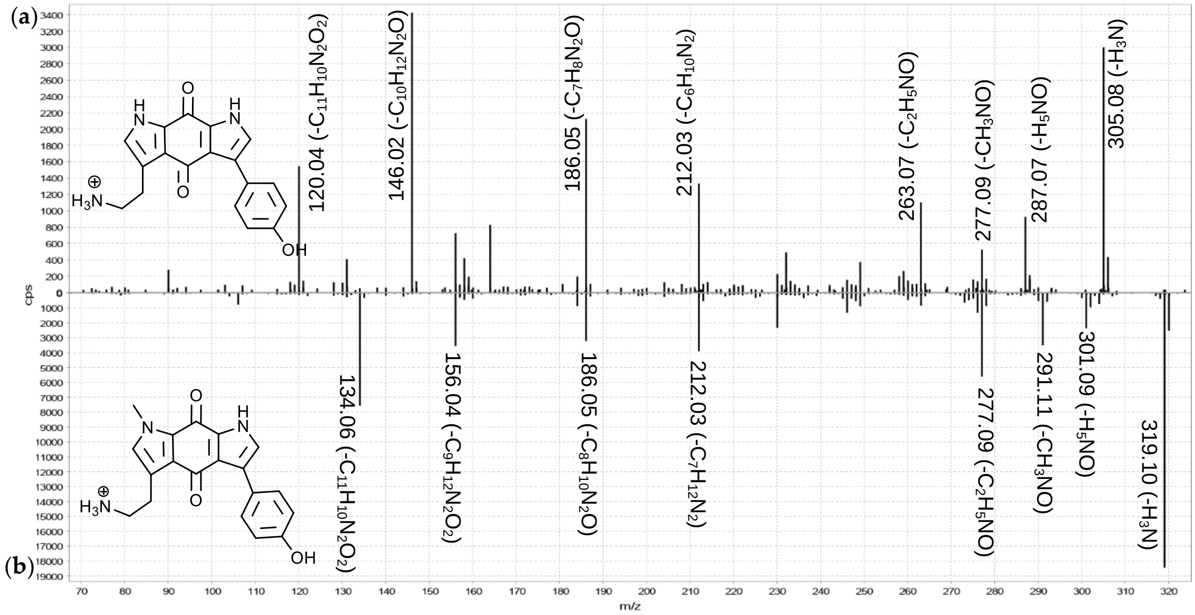

S3.21: MS[2] spectra of the 322.1 (**a**) and 336.1 Da (**b**) nodes in group O, possibly representing zyzzyanones.

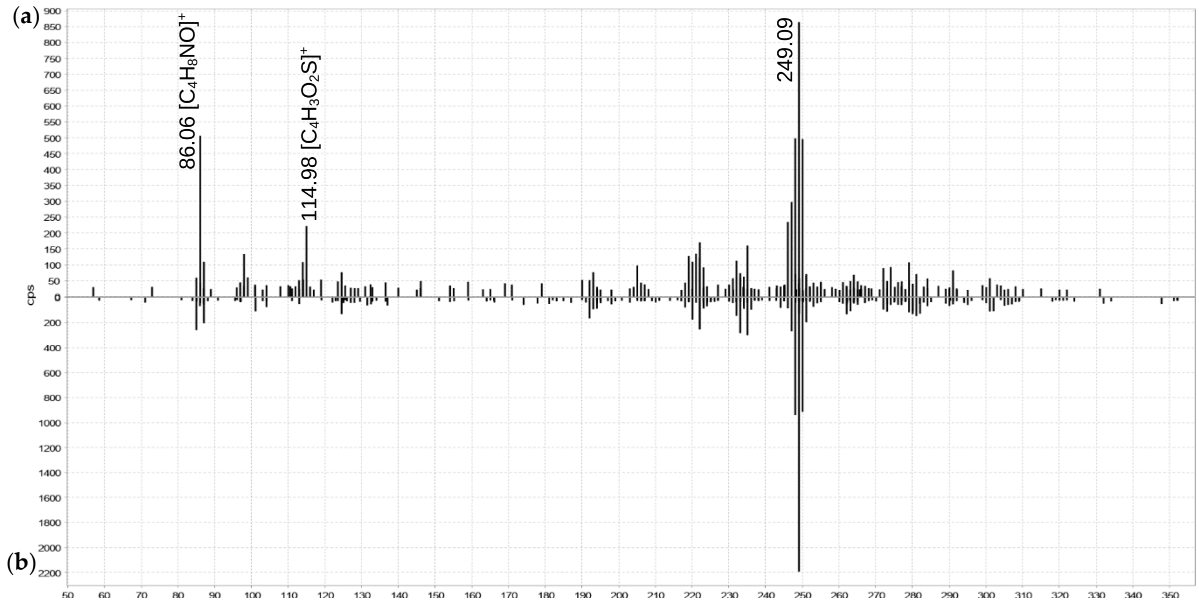

S3.22: MS^2 spectra of the 479.1 Da (**a**) and 507.2 Da (**b**) nodes from group O.
